# Constructing Seamless Interfaces for Ultrastable Flexible Supercapacitors

**DOI:** 10.1002/advs.75418

**Published:** 2026-04-21

**Authors:** Keyi Dong, Zefeng Yan, Weiyang Tang, Quanhu Sun, Jiaxin Yang, Yan Tan, Yu Wang, Tian Lv, Tao Chen

**Affiliations:** ^1^ Shanghai Key Lab of Chemical Assessment and Sustainability School of Chemical Science and Engineering Tongji University Shanghai P. R. China

**Keywords:** anchored interfacial polymerization, flexible, heterogeneous polyelectrolytes, in situ polymerization, supercapacitor

## Abstract

Flexible supercapacitor (SC) represents a promising power source for wearable electronics, but its cycling and flexible stabilities are currently limited by the weak interaction at the electrode/electrolyte interface. We construct a covalent electrode/electrolyte interface by anchored interfacial polymerization and a topologically entangled electrolyte/electrolyte interface by in situ polymerization in one SC. The interfacial toughness and adhesion strength between electrode and electrolyte reach 2965.3 J m^−2^ and 241.6 kPa, respectively, which are two orders and one order of those assembled by the traditional stacking approach. The seamless interfaces efficiently promote ion transport ability among them, which provides the SC with a three‐time enhancement of specific capacitance, compared with the device by the DS method. The developed SC exhibits high capacitance retention of 89.5% for 10^5^ charge/discharge cycles and 96.2% after folding for 10^5^ times, indicating outstanding stability of structure and performance. The demonstrated seamless interfacial engineering can be easily expanded in the field of other flexible electronics.

## Graphical abstract

Seamless electrode/electrolyte and electrolyte/electrolyte interfaces with boosted interfacial toughness and adhesion are constructed by anchored interfacial polymerization and in situ polymerization, respectively. The achieved supercapacitor exhibits ultrahigh capacitance retention over 100,000 times of charge/discharge or folding deformation.

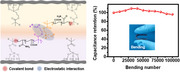

## 1 Introduction

The rapid development of wearable electronics (e.g., intelligent sensors, electronic skin, and brain−computer interface) requires high−performance flexible energy storage systems with excellent stability of structures and properties [1, 2, 3]. As one of the most important energy storage devices, electrochemical supercapacitors (SCs) with high power density, rapid charging ability, and long cycle life that can be easily fabricated into flexible devices, represent a promising candidate for powering portable and flexible electronics [4, 5, 6]. Generally, flexible SCs are often constructed by assembling substrate, electrode, separator, and polymeric gel electrolyte together through a direct stacking (DS) approach [7], and many achievements (such as one−dimension fibrous, two−dimension thin−film, and in−plane interdigital devices) have been achieved so far [8, 9, 10]. Physical interfacial contact between electrode and electrolyte was formed by the DS technique, because it is difficult for the polymeric gel electrolytes with high viscosity to infiltrate into the micropores of electrode materials. The generated electrode/electrolyte interface with relatively weak interaction is not conducive to efficient diffusion and transport of ions, which easily cause interlayer slippage and irreversible delamination as the devices were under mechanical deformations (e.g., bending, folding, or stretching), resulting in serious performance degradation and/or loss of their functions [11, 12, 13, 14]. As a result, almost no flexible SCs that can withstand hundreds of thousands of repeated bending or folding deformations were reported so far.

To optimize the interfacial contact between electrode and electrolyte, in situ polymerization of gel electrolyte has been explored to sandwich two pieces of electrodes with polymer electrolyte in between, by which the electrode materials can be well infiltrated with pre−polymer solution and further cross−linked by the formed polymeric chains of gel electrolyte [12, 15, 16]. Mechanical interlocking and topological entanglement have also been constructed to expand the contact area between gel electrolyte and electrode for energy storage devices with good flexibility [11, 17, 18]. In addition, some efforts have been made to introduce Van der Waals interactions, hydrogen bonds, and electrostatic interactions between the electrode and polymer electrolyte of SCs to enhance their interfacial interaction [19, 20, 21, 22]. However, the above approaches cannot change the intrinsic characteristics of the physically contacted interface between electrode and electrolyte, the phase separation between which cannot be prevented after thousands of bending deformations. Covalently contacted interfaces have been constructed via chemical reactions between electrode and electrolyte, as well as in situ polymerization of monomers anchored on electrode materials [13, 23, 24], but the demonstrated flexible SCs showed relatively poor electrochemical performance. Another great challenge of flexible SCs is their rapid self−discharge rate derived from their intrinsic energy storage mechanisms of ion absorption or Faraday reactions. In this regard, we proposed an efficient strategy by constructing a bilayer polycation/polyanion polymer electrolyte to compress the self−discharge rate of flexible SCs, which was realized by the strong interaction between the charged polyelectrolytes and the ions accumulated on/in electrode [25, 26, 27, 28, 29]. Therefore, it will be greatly meaningful to develop ultrastable flexible SCs with slow self−discharge for practical applications in the fields of various portable electronics.

We here demonstrate a type of ultrastable flexible SCs with superlong cycling and repeated folding stabilities, which is enabled by designing multiple seamless interlocked interfaces via anchored interfacial polymerization (AIP) between electrode and electrolyte, as well as in situ polymerization (ISP) between two layers of electrolytes. On the one hand, the silane coupling agent modified electrodes are functionalized with hydrophobic initiators, which can generate radicals under UV radiation to initiate polymerization of the anionic monomers in the prepolymer solution of electrolytes, referred as AIP. The resultant electrode/electrolyte interface with strong covalent interactions provides it with ultrahigh robust interfacial toughness of 2965.3 J m^−2^ and tough interfacial adhesion of 241.6 kPa between electrode and electrolyte. On the other hand, a cationic monomer solution is in situ polymerized on top of the first pre‐polymerized anionic electrolyte, through which a polymeric entangled interface with strong electrostatic interactions is constructed between two layers of polymer electrolytes. The strong interfacial interactions of the seamlessly interlocked multiple interfaces endow the assembled symmetric supercapacitor (CNTs as electrodes) with ultralong cycling charge/discharge stability (capacitance retention of 89.5% for 10^5^ cycles) and ultrastable flexibility (96.2% of the initial capacitance after folding for 10^5^ times). Through this approach, the flexible SC that exhibits high specific capacity (199.4 mF cm^−2^), energy density (84.2 µWh cm^−2^), and power density (581.6 µW cm^−2^) is further developed by using active carbon (AC) as negative and manganese dioxide (MnO_2_) as positive. The existing strong electrostatic repulsions between charged polyelectrolytes and movable ions enable the resultant flexible SCs with a super low self−discharge rate (0.038 V h^−1^).

## 2 Results and Discussion

### 2.1 Designing Concept and Preparation of Supercapacitor with Multiple Seamless Interlocked Interfaces

**FIGURE 1:**
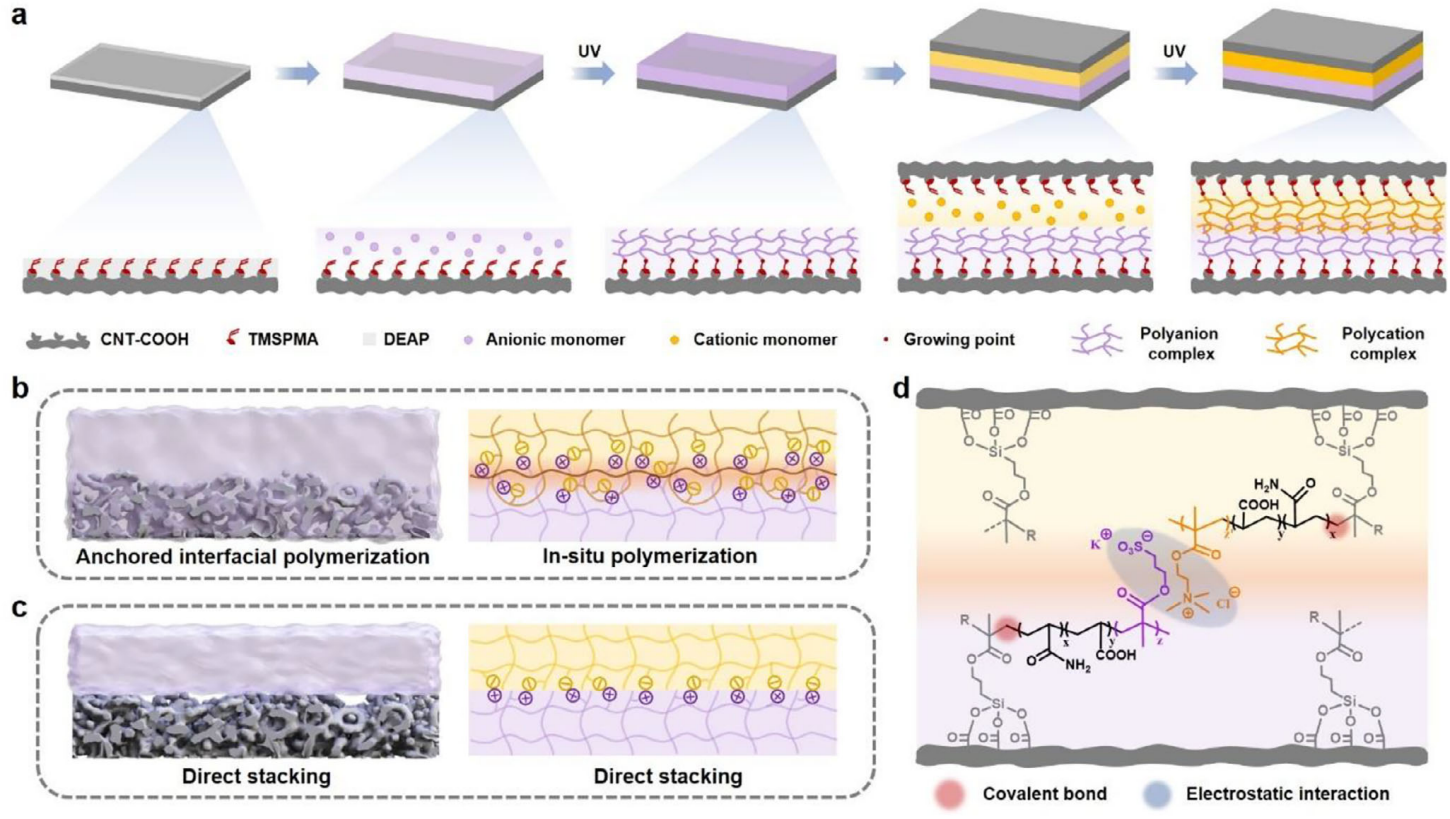
Schematics showing the fabrication and structure of SC with multiple seamless interfaces. (a) Schematic diagram illustrating the detailed process to fabricate SC by AIP+ISP method. (b) The schematics of electrode/electrolyte interface by the AIP approach and the electrolyte/electrolyte interface formed through ISP method, respectively. (c) The schematics of electrode/electrolyte interface and electrolyte/electrolyte interface formed by DS assembling process. (d) Schematic diagram to show the interactions at multiple interfaces inside the SC by AIP and ISP method, including the covalent bond between the electrode and electrolyte, as well as the electrostatic interaction between PAC and PCC electrolytes.

The design concept was schematically illustrated in Figure 1a to achieve ultrastable flexible SCs through AIP and ISP approaches. To start with, a silane coupling agent of 3−(trimethoxysilyl)propyl methacrylate (TMSPMA) was first grafted on the carboxylated CNTs (CNTs−COOH) film, which was confirmed by X−ray photoelectron spectroscopy (XPS, Figure S1). From the energy dispersive spectroscopy (EDS) mapping (Figure S2), it can be seen that the elements of oxygen (O) and silicon (Si) in the CNTs−COO−TMSPMA film are distributed uniformly. After that, the initiator of 2,2−diethoxyacetophenone (DEAP) dissolved in ethyl acetate was directly dropped on the surface of the CNTs−COO−TMSPMA film, during which process the contact angle (Figure S3) rapidly decreased from 5.05° to 0° within 15 s with volatilization of ethyl acetate, formed a uniform initiator layer. While the water contact angle on CNTs−COO−TMSPMA film coated with initiator layer was about 71.96°, suggesting hydrophobic of initiator layer. As the prepolymer aqueous solution of polyanion complex (PAC) was spread on CNTs−COO−TMSPMA film coated with initiator layer, the initiator of DEAP generated radicals and induced the polymerization of TMSPMA on the surface of CNTs−COO−TMSPMA film and monomers in prepolymer solution with UV radiation, resulting in seamless covalently connected interface between CNTs and PAC electrolyte (Figure 1b). The prepared PAC−grafted CNTs were covered on the prepolymer solution of polycation complex (PCC) on another piece of CNTs−COO−TMSPMA film, followed by polymerization (Figure 1a) to achieve the SC device. The second prepolymer solution can penetrate into the first PAC networks for further polymerization, achieving a topologically entangled electrolyte/electrolyte (PAC/PCC) interface (Figure 1b). In contrast, there were obvious gaps (Figure 1c) at the interface between electrode and electrolyte in the SC assembled by the traditional DS process, because only a weak Van der Waals' force existing between the electrode and polymer electrolyte. Figure 1d schematically showed the strong interactions among constructed multiple interfaces inside the SC device fabricated through AIP and ISP method in detail.

### 2.2 Structures of the Multiple Interfaces and Ion Transport Performance

**FIGURE 2:**
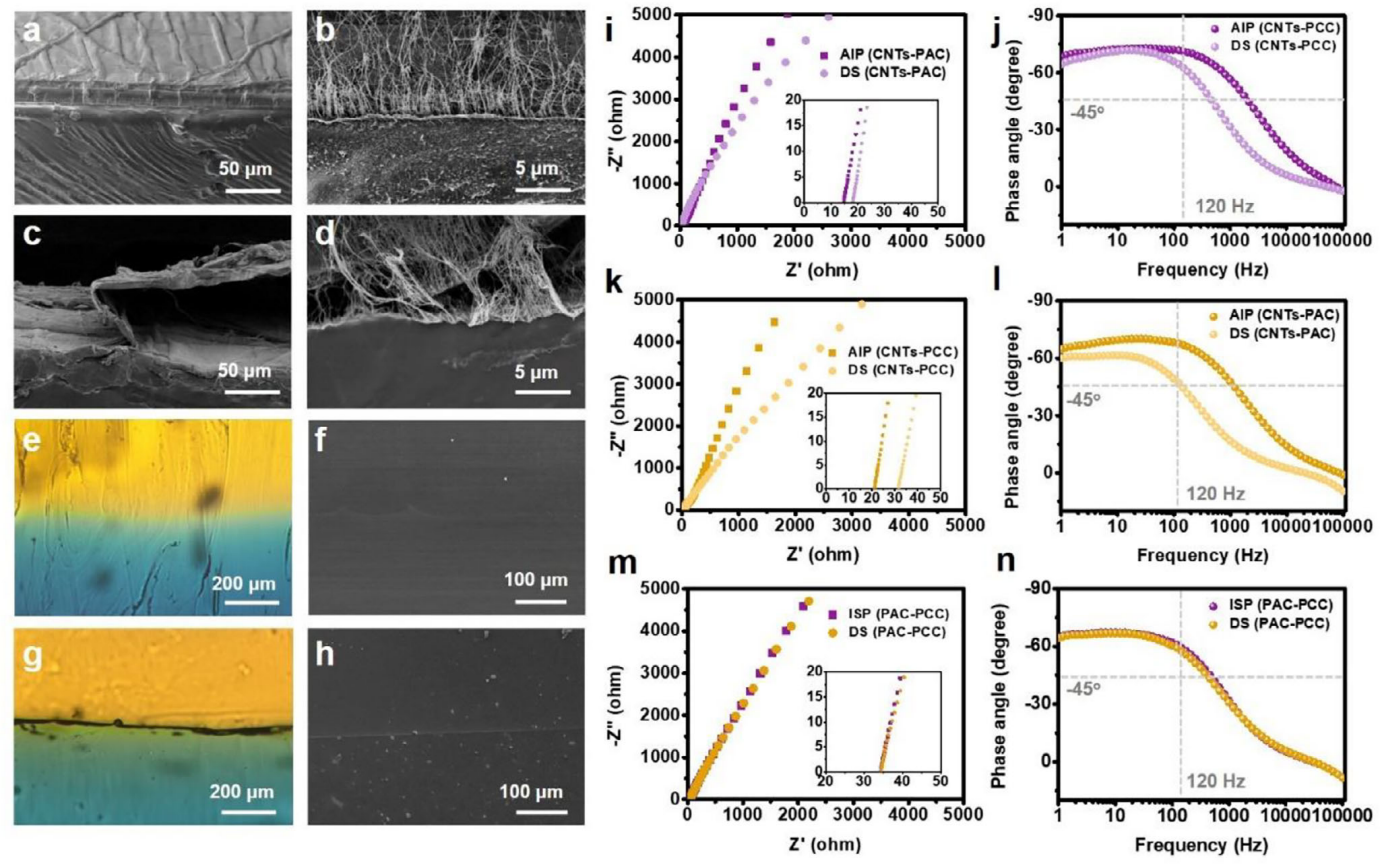
Structures and electrochemical performance of interfaces of electrode/electrolyte and electrolyte/electrolyte. Cross−sectional SEM images of electrode/electrolyte interfaces through (a,b) AIP method and (c,d) DS process. Optical micrographs and cross−sectional SEM images of electrolyte/electrolyte interfaces via (e,f) ISP method and (g,h) DS approach. (i) Nyquist plots and (j) Bode plots of CNT−COO−TMSPMA films with PAC formed through AIP and DS methods. (k) Nyquist plots and (l) Bode plots of CNTs−COO−TMSPMA films with PCC formed through AIP and DS methods. (m) Nyquist plots and (n) Bode plots of bilayer heterogeneous electrolyte via ISP and DS methods.

The interfacial structure between the polymer electrolyte and electrode (or electrolyte) was characterized by scanning electron microscope (SEM) and fluorescence microscope. As Figure 2a showed, the contact interface between CNTs electrode and the polymer electrolyte was seamlessly connected without any gap for the sample fabricated by the AIP method. There was a compact CNTs network left on the hydrogel electrolyte (Figure 2b), even teared CNTs from the electrolyte. In contrast, an obvious non−contact interface (gap) between CNTs electrode and the hydrogel electrolyte can be seen from Figure 2c for the sample prepared by DS process, and the CNTs film was easily peeled from the hydrogel electrolyte (Figure 2d). For the interface between PAC and PCC hydrogel electrolytes achieved by ISP method, ideal seamless interface between them (Figure 2e,f) was formed, with penetration of the second prepolymer solution into the first polymerized hydrogel electrolyte. Meanwhile, the EDS mapping further revealed two layers of PAC and PCC well fused together with each other at the interface (Figure S4). For comparison, it is also inevitable to leave some interfacial gap (Figure 2g,h) between two layers of PAC and PCC with using DS assembling approach, though the polymer hydrogels are more adhesive and flexible than CNTs−based or other electrodes.

The electron/ion transport ability at the covalently linked electrode/electrolyte interface was evaluated via electrochemical impedance spectroscopy (EIS), where the polymer electrolyte was sandwiched between two platinum plates. As for CNTs−COO−TMSPMA films with PAC (CNTs−PAC), the series resistance was 14.7 and 18.1 Ω for the films fabricated by methods of AIP and DS, respectively (Figure 2i). The lower resistance and more vertical slope of the seamless interface than that of DS film indicated that the CNTs−PAC film by the AIP approach possessed a rapid ionic migration and ideal capacitive characteristic. At the phase angle of −45° (Figure 2j), the measured frequencies of CNTs−PAC formed by methods of AIP and DS were 2153 and 464 Hz, respectively, which revealed the ISP‐formed interface having more capacitive behavior and the DS‐formed interface with more resistive behavior [30, 31, 32]. At the frequency of 120 Hz (Figure 2j), the CNTs−PAC through AIP method possessed a lower phase angle of −71.7° than the CNTs−PAC by DS process (−64.5°), once again suggesting the ideal capacitive behavior of CNTs−PAC with seamless interface. Similarly (Figure 2k,l), the CNTs−COO−TMSPMA‐grafted PCC (CNTs−PCC) through AIP method exhibited lower resistance (21.3 Ω), higher frequency (1211 Hz) at a phase angle of −45° and lower phase angle (−67.7°) at 120 Hz than those of film by DS approach (31.7 Ω, 146 Hz and −47.0°). For the bilayer heterogeneous hydrogel electrolytes (PAC−PCC), almost no obvious differences were observed for both Nyquist plots and Bode plots (Figure 2m,n), whether the bilayer electrolyte was prepared by ISP or DS approach, which can be ascribed to the excellent adhesivity between the two types of hydrogel electrolytes and the existing electrostatic attraction between PAC and PCC. As a result, the ionic conductivities of PAC−PCC electrolytes through ISP (4.7 mS cm^−1^) and DS (4.6 mS cm^−1^) were very close. However, the adhesive force between two layers of hydrogel electrolytes must have a large difference because of the different interfacial interaction derived from two fabricating processes, which will be discussed infra. In addition, galvanostatic intermittent titration technique (GITT) tests were employed to quantify the ion diffusion coefficients of the bilayer polymer electrolytes in SCs with using CNTs electrodes. As Figure S5 showed, the ion diffusion coefficients of PAC−PCC electrolyte in the SC fabricated by AIP+ISP were about 2.06 × 10^−6^ and 2.30 × 10^−6^ cm^2^ s^−1^ during charge and discharge process, respectively, much higher than the devices fabricated by other methods. The results revealed that the seamless contact interface can efficiently enhance the ionic transport ability and capacitive behavior between the electrode and electrolyte, which is favorable for building high−performance flexible SCs with superior structural stability.

### 2.3 Electrochemical Performance of SCs

**FIGURE 3:**
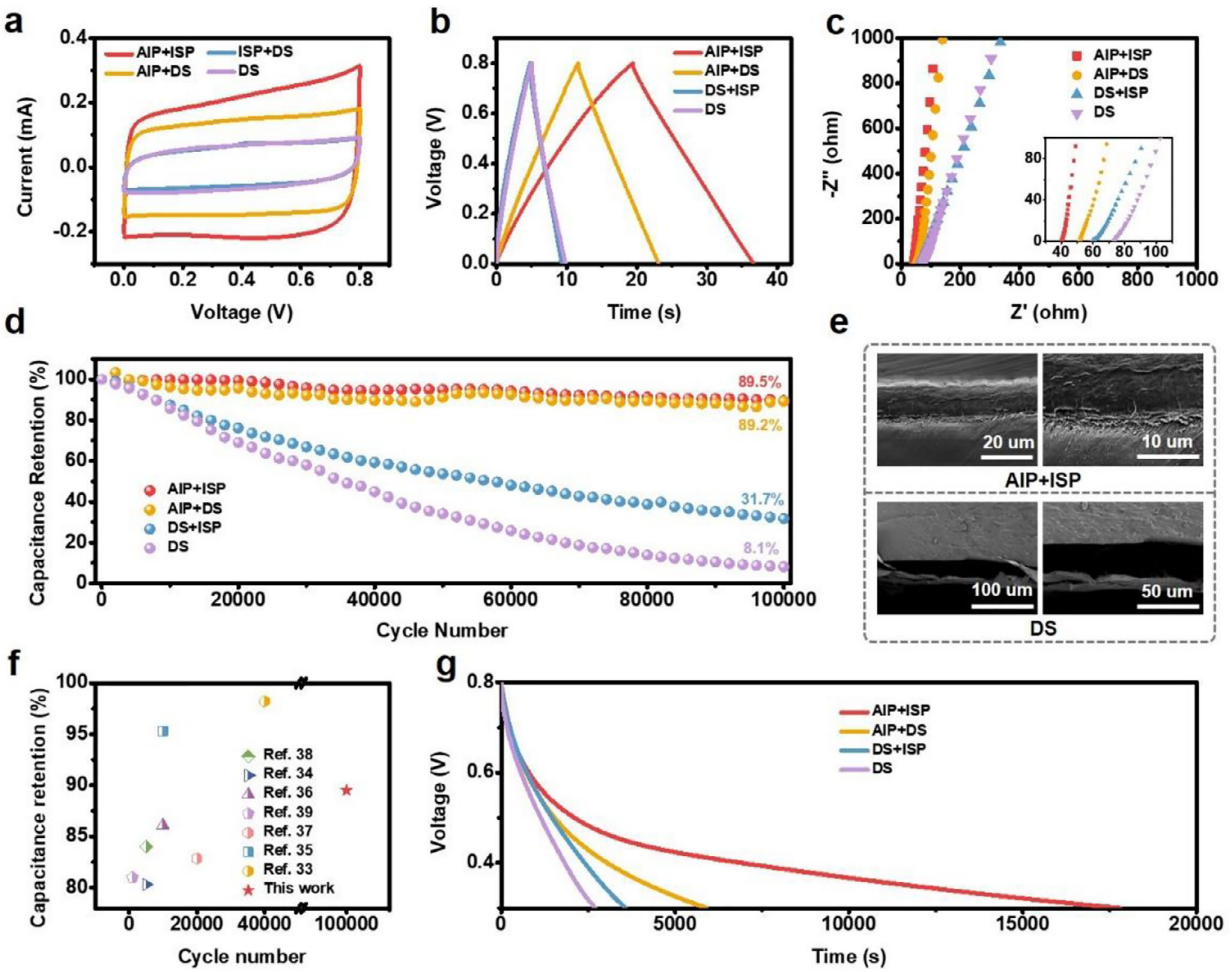
Electrochemical performance of SCs assembled by different methods. (a) CV curves at a scan rate of 100 mV s^−1^, (b) GCD curves at a current density of 0.2 mA cm^−2^, (c) Nyquist plots, and (d) cyclic performance of SCs assembled by different methods. (e) Cross−sectional SEM images of electrode/electrolyte interface fabricated by AIP+ISP and DS methods after 100 000 charge−discharge cycles. (f) Comparison of capacitance retention of our supercapacitor assembled by AIP+ISP method after 100 000 charge−discharge cycles with other reported results. (g) Self−discharge curves of SCs assembled by different methods.

A series of SCs were constructed by using bilayer hydrogel electrolytes of PAC−PCC and CNTs−based electrodes through different assembling processes, including AIP+ISP, AIP+DS, DS+ISP, and DS approaches. The details of fabricating process were described in the experimental section. As shown in Figure 3a,b, all the cyclic voltammetry (CV) curves possessed nearly rectangular shapes, and all the galvanostatic charge−discharge (GCD) curves exhibited symmetric triangular shapes, revealing these SCs with ideal capacitive behavior. Calculated from GCD curves, the specific capacitances of SCs assembled by DS+ISP and DS methods were almost the same (1.2 mF cm^−2^), which were further enhanced to 2.9 and 4.4 mF cm^−2^ for the devices assembled by AIP+DS and AIP+ISP processes, respectively. The highest capacitance of SC by AIP+ISP can be attributed to its multiple seamless contact interfaces, which can evidently reduce the interfacial resistances and efficiently facilitate ions transport across the interfaces. The EIS measurements (Figure 3c) further confirmed that the series resistances of SCs assembled by DS, DS+ISP, AIP+DS, and AIP+ISP approaches gradually decreased from 73.4 Ω to 59.7, 51.4, and 39.8 Ω, with increasing the number of seamless interfaces. The SC by AIP+ISP exhibited the highest slope within low−frequency region, which revealed a lower surface diffusion resistance than the devices assembled by the other three approaches. Furthermore, all the CV and GCD curves (Figure S6) of SC based on AIP+ISP well maintained their shapes at different scan rates and different current densities, respectively, indicating excellent rate performance.

Benefiting from the seamless compact interfaces, the SC made by AIP+ISP method exhibited a high capacitance retention of 89.5% (Figure 3d) after 100 000 charge/discharge cycles, which was over two times of the device assembled by DS+ISP (31.7%) and ten times of the device fabricated by DS process (8.1%). It should be noted that the SC made by AIP+DS showed a capacitance retention of 89.2% for 100 000 charge/discharge cycles, very closed to the device by AIP+ISP. The results revealed that the constructed seamless electrode/electrolyte interface can efficiently enhance the ions transport ability and structural stability between them, providing the resultant SCs with higher specific capacitance and longer cycling lifetime than the device by DS assembling approach. From the cross−sectional SEM images shown in Figure 3e, it could be clearly seen that the seamless interface between electrode and electrolyte fabricated by AIP almost unchanged after 100 000 charge/discharge cycles compared with its original states (Figure 2a), which validated the feasibility of our design concept. While for the SC by DS assembly, the relative weak interfacial interaction between electrode and electrolyte cannot bear such long charge/discharge cycles. As a result, the two layers will easily crack and separate with each other (Figure 3e), causing serious degradation of electrochemical performance. The cycling numbers over 100 000 times of our SCs are almost the highest value, compared with other reported results (Figure 3f) [33, 34, 35, 36, 37, 38, 39].

The aim of designing a seamless bilayer hydrogel electrolyte of PAC and PCC is to reduce the self−discharge rate of SC, derived from the energy storage mechanism of surface ions absorption/adsorption or Faraday reaction. Our previous work demonstrated that the existing strong electrostatic repulsion between charged polyelectrolytes and the same charge type of ions accumulated in/on electrode can efficiently restrict the redistribution of ions in a charged SC [25, 26, 27, 28, 29]. Figure 3g showed that the self−discharge time (from 0.8 to 0.3 V) of SCs prepared via AIP+ISP, AIP+DS, DS+ISP, and DS+DS method was 17811, 5916, 3587, and 2709 s, respectively. These results indicated that both electrode/electrolyte and electrolyte/electrolyte interfaces played indispensable roles in compressing the self−discharge rates of SCs. The perfect electrode/electrolyte interface allowed more ions aligned in/on electrodes, which making them generating much strong electrostatic repulsion with the charged polyelectrolytes to prevent these ions redistribution. Meanwhile, the intrinsic built−in electric field at polycation/polyanion interface formed through DS would hinder the migration of ions during the charging process, which will accelerate the shedding of adsorbed ions on the electrodes during the self−discharge process. In contrast, the in situ formed electrolyte/electrolyte interface with numerous disordered electric fields could weaken the adverse effect of built−in electric field caused by the bilayer electrolyte of PAC−PCC (Figure S7). We have systematically optimized the electrochemical performance (Figures S8–S10) of SCs through adjusting the concentration of monomers and the thickness of hydrogel electrolytes, and the molar concentrations ratio of 8:2:2 (acrylamide: acrylic acid: methacryloxyethyltrimethyl ammonium chloride or 3−sulfopropyl methacrylate potassium salt). PAC‐PCC with total thickness of 800 µm were selected to achieve supercapacitor devices with balanced capacity, impedance and self−discharge rate for further investigation.

### 2.4 Mechanical Flexibility and Stability of SCs

**FIGURE 4:**
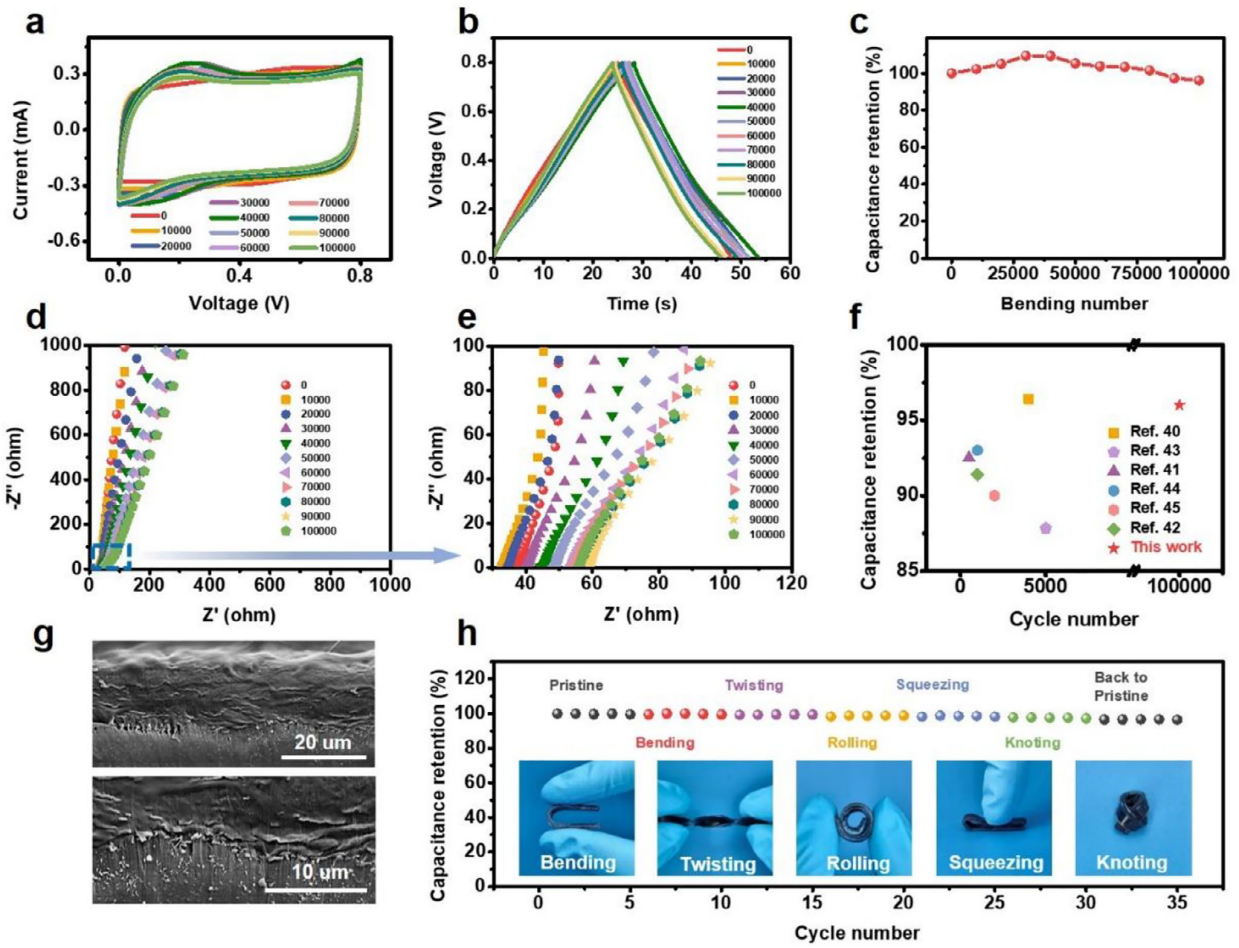
Flexibility of the supercapacitor assembled by AIP+ISP method. (a) CV curves at a scan rate of 100 mV s^−1^, (b) GCD curves at a current density of 0.2 mA cm^−2^, and (c) capacitance retention of the supercapacitor being folded for 100 000 times. (d,e) Nyquist plots of the supercapacitor after 100 000 bending numbers. (f) Comparison of flexibility of our supercapacitor assembled by AIP+ISP method with other reported results. (g) Cross−sectional SEM images of electrode/electrolyte interface fabricated through AIP+ISP after being folded for 100 000 times. (h) Capacitance retention of our supercapacitor under various mechanical deformations.

Besides ultralong cyclic life and slow self−discharge rate, the SC with multiple seamless interfaces also exhibited superflexibility. The CV and GCD curves variated very slightly (Figure 4a,b), and the capacitance retention of the supercapacitor reached 96.2% even after being bent to 180° (fold) for 100 000 times (Figure 4c). The slightly performance degradation of SC with multiple seamless interfaces can be ascribed to the increase of series resistance (from 37.3 to 55.7 Ω) and the slight decrease of surface ions diffusion ability (reduction of slope with low frequency region) after folding for 100 000 times (Figure 4d,e). Compared with other reported flexible SCs, the flexible stability and bending cycles of our newly−developed SC with multiple seamless interfaces are the highest ranges (Figure 4f) [40, 41, 42, 43, 44, 45]. The outstanding flexibility and stability of the developed SC can be attributed to the ultrastable multiple interfaces with seamless structures, which made them contacting tightly (Figure 4g) for hundreds of thousands of bending cycles. Furthermore, the SC with multiple seamless interfaces can well maintain its original performance under various mechanical deformations, including bending, twisting, rolling, squeezing, and knotting (Figure 4h), which showed great potential for further practical application under complex conditions.

### 2.5 Interfacial Toughness and Adhesion Strength Between Electrode and Electrolyte in SCs

**FIGURE 5:**
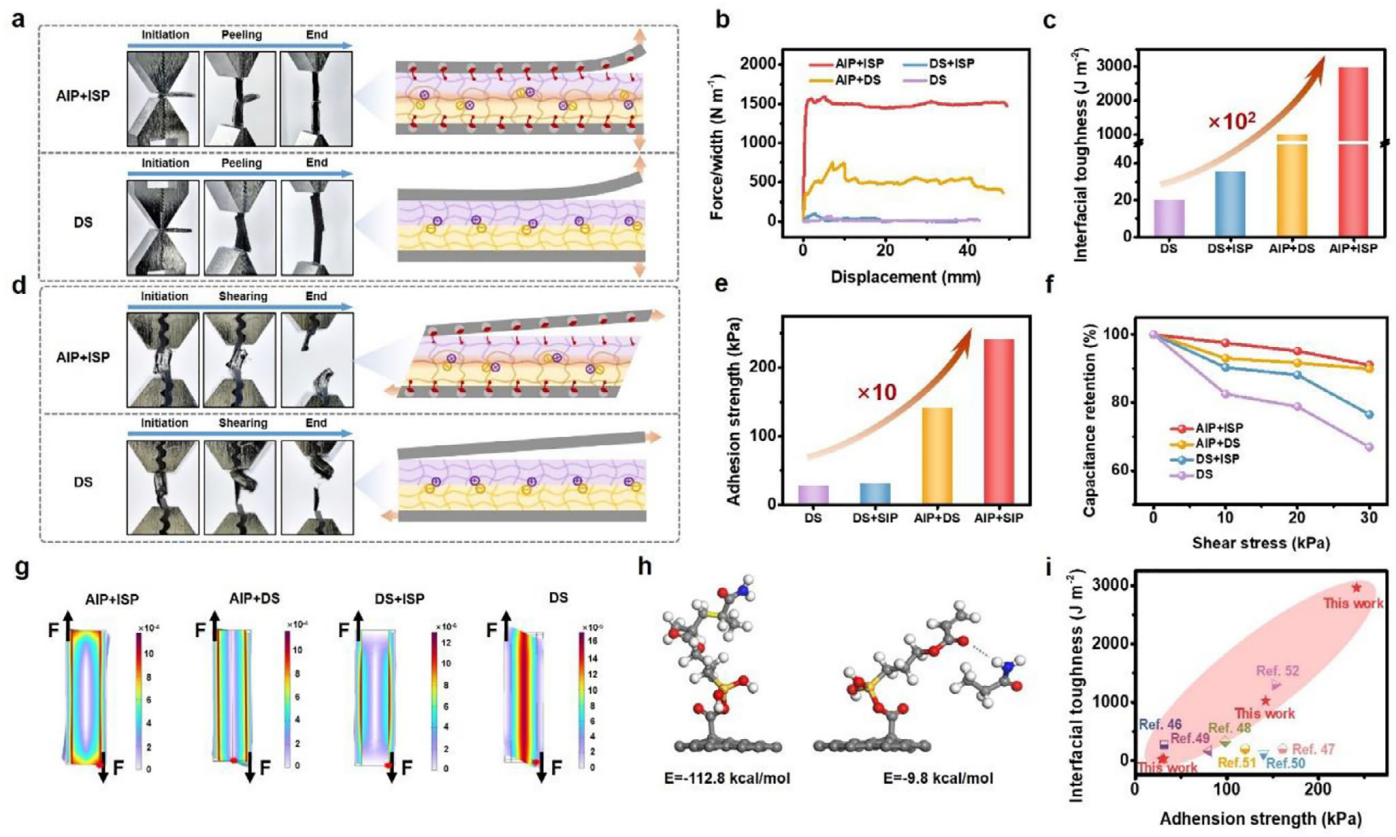
The characterization of the interfacial toughness and adhesion strength of SCs with different interface. (a) Optical images and Schematic diagram of the 180° peeling process for SCs assembled by AIP+SIP and DS. (b) The 180° peeling force per width versus displacement curves of the SCs. (c) Interfacial toughness of the SCs. (d) Optical images and schematic diagram of the lap−shear process for SCs assembled by AIP+SIP and DS. (e) Adhesion strength of the SCs. (f) Capacitance retention of SCs with different interface under shear stress. (g) Stress distribution of the SCs with different interfaces under shear stress was simulated by FEM (unit: N m^−2^). (h) Binding energy between CNTs−COO−TMSPMA and a unit of PAM after AIP and DS was obtained by DFT calculation. (i) Comparison of interfacial toughness and adhesion strength of our SCs with other reported results.

To insightfully reveal the enhanced mechanism of multiple seamless interfaces for the structural stability of SCs, 180° peeling tests and lap−shear measurements were performed to evaluate the interfacial toughness and adhesion at various interfaces. Figure 5a and Figure S11 showed digital photographs of SCs assembled by different approaches for 180° peeling tests, according to which the damage mechanisms for various types of SCs can be revealed. As for devices based on AIP+ISP method, the damage first occurred at the electrode/electrolyte interface, which indicated the ISP‐formed electrolyte/electrolyte interface possessed a stronger binding force (with crosslinked macromolecular network, electrostatic interaction, and hydrogen bonds) than that of the electrode/electrolyte interface by AIP (only covalent bonds between hydrogel molecules and partial sites of CNTs). The average peeling force and interfacial toughness between electrode and electrolyte were as high as 1489.8 N m^−1^ and 2965.3 J m^−2^ (Figure 5b,c), respectively. While for SC with using AIP+DS method, the damage occurred at the interface between PAC and PCC hydrogel electrolytes, due to their weak interfacial interactions derived from adhesion force and electrostatic interaction. The peeling force and interfacial toughness between two DS hydrogel electrolytes were 508.9 N m^−1^ and 1024.6 J m^−2^, respectively. In SCs constructed by both DS+ISP and DS methods, the peeling force and interfacial toughness between CNTs electrode and the hydrogel electrolyte were almost the lowest among the four approaches, due to inefficient interfacial contact and a lack of interfacial interaction between them. The XPS depth profiling (Figure S12) showed that S 2p of PAC and N 1s of PCC could still be detected inside the peeled electrodes fabricated by AIP approach, which revealed the polyelectrolytes existed throughout the entire electrodes. In contrast, there was almost no residual electrolyte on the peeled electrodes prepared by DS method, due to the weak Van der Waals interactions between the electrode and polymer electrolyte.

The adhesion strengths at multiple interfaces in SCs assembled by different approaches were evaluated through lap−shear tests, and the damage mechanisms were schematically concluded (Figure 5d and Figure S13). The adhesion strength of AIP−derived electrode/electrolyte interface was about 241.6 kPa, nearly ten times of that made by DS process (Figure 5e and Figure S14). The dependence of capacitance on the shear stress was also investigated. As Figure 5f and Figure S15 showed, the capacitance of SCs assembled through DS+SIP and DS rapidly decreased with increase of shear stress to 30 kPa, while the devices based on AIP+ISP and AIP+DS can maintain about 90% of their original capacitance at the high shear stress of 30 kPa, due to the very tiny slippage occurring at the interfaces. The interfacial toughness and adhesion strength between electrode and electrolyte based on AIP+ISP method still maintained 1143.5 J m^−2^ and 155.6 kPa, respectively, even after 100 000 charge/discharge cycles (Figure S16). The superior stabilities under 180° peeling and high shear stress enabled by the multiple seamless interfaces make flexible supercapacitors having the ability to withstand any unpredictable mechanical deformations in practice. For comparison, the interfacial toughness and adhesion strength between electrode and polymer electrolyte formed by conventional in situ polymerization were only 117.5 J m^−2^ and 31.2 kPa (Figure S17), much lower than the electrode/electrolyte interface constructed by AIP+ISP method. From the stress distribution maps (Figure 5g) constructed by the finite element method (FEM), the interface constructed by AIP+ISP in an SC possessed the largest shear stress, compared with other assembling approaches. To further understand the interfacial interaction between electrode and electrolyte, the binding energy between a simplified model containing CNT−COO−TMSPMA and one unit of PAM (via AIP or DS) was conducted by density functional theory (DFT) calculation. The results (Figure 5h) revealed that the dissociation energy of covalent bond between CNT−COO−TMSPMA and PAM reached −112.8 kcal mol^−1^, over ten times stronger than that of hydrogen bonds and van der Waals forces (−9.8 kcal mol^−1^) between them. As a consequence, the interfacial toughness and adhesion strength between CNTs electrode and hydrogel electrolyte in our SCs were much more remarkable than those between the hydrogel and the substrate in other reported results (Figure 5i) [46, 47, 48, 49, 50, 51, 52].

### 2.6 Universal Availability of Multiple Seamless Interfaces for Superflexible High‐Performance SCs

To validate the universal availability of the concept for other electrode materials, we further constructed an asymmetrical SC with using a positive electrode of manganese dioxide modified CNTs (MnO_2_/CNTs) and a negative electrode of active carbon−coated CNTs (AC/CNTs). The uniform AC layer with a thickness of about 80 µm (Figure S18a) was blade−coated on the surface−modified CNTs film to obtain a negative electrode of AC/CNTs, and MnO_2_ with a thickness of about 50 µm (Figure S18b) was electrochemically deposited on CNTs substrate. The achieved MnO_2_/CNTs and AC/CNTs were confirmed from Raman spectra (Figure S19). Both of MnO_2_/CNTs positive electrodes and AC/CNTs negative electrodes exhibited ideal capacitive behavior and outstanding rate performance (Figures S20 and S21). The specific capacitances of individual MnO_2_/CNTs and AC/CNTs reached 457.1 and 353.2 mF cm^−2^, respectively.

**FIGURE 6:**
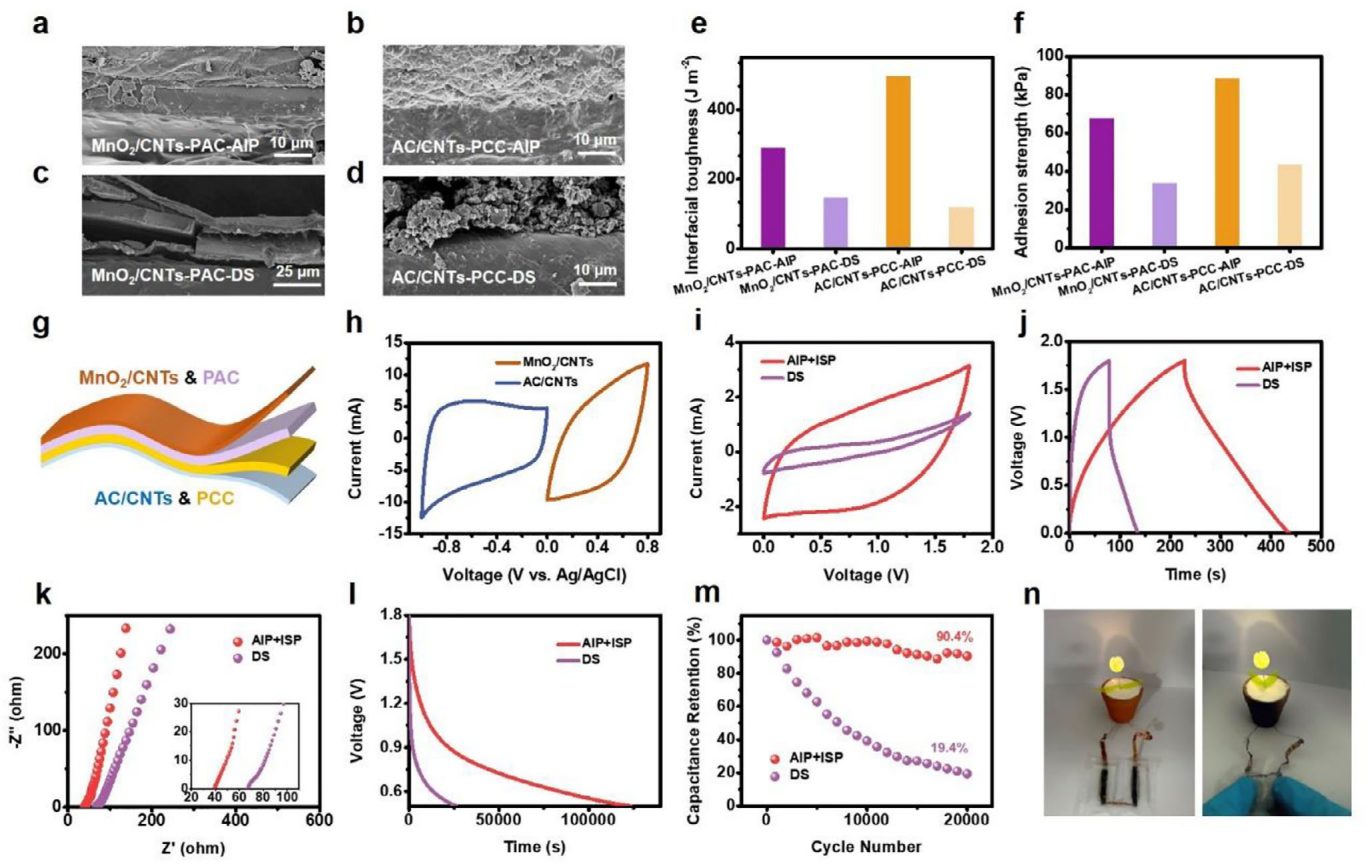
Electrochemical and interfacial performance of asymmetric supercapacitor assembled by AIP+ISP. Cross−sectional SEM images of the interface between PAC and MnO_2_/CNTs−T, as well as between PCC and AC/CNTs−T through (a,b) AIP+ISP method and (c,d) DS. (e) Interfacial toughness and (f) adhesion strength of the electrodes/electrolytes interface through AIP+ISP method and DS. (g) Schematic diagram of an asymmetric supercapacitor. (h) CV curves of individual MnO_2_/CNTs and AC/CNTs electrodes measured in a three−electrode system at the scan rate of 20 mV s^−1^. (i) CV curves at the scan rate of 100 mV s^−1^, (j) GCD curves at the current density of 1.3 mA cm^−2^, (k) Nyquist plots, (l) self−discharge curves, and (m) cyclic performance of supercapacitor assembled by AIP+ISP and DS. (n) Optical images of supercapacitor in series powering a flower‐shaped diode (working voltage of 3 V) under flat state and bent to 180°.

The MnO_2_/CNTs and AC/CNTs were also modified with TMSPMA (Figure S22), the resultant electrodes were named as MnO_2_/CNTs−T and AC/CNTs−T, respectively. The XPS spectra (Figure S23) of C 1s and Si 2p of the MnO_2_/CNTs−T and AC/CNTs−T electrodes confirmed the TMSPMA molecules were successfully grafted on relative electrodes, and all the elements were uniformly distributed in the modified electrodes (Figures S24 and S25). From Figure 6a,b, the electrode/electrolyte interface formed through AIP was seamless connected between PAC and MnO_2_/CNTs−T (MnO_2_/CNTs−T−PAC) (also between PCC and AC/CNTs−T (AC/CNTs−T−PCC)), but huge creaks existed at electrode/electrolyte interface formed by DS approach (Figure 6c,d). 180° peeling tests (Figure S26) showed that the interfacial toughness of MnO_2_/CNTs−T−PAC and AC/CNTs−T−PCC prepared by AIP method was 291.8 and 496.7 J m^−2^, respectively, while those prepared by DS were only 148.9 and 120.0 J m^−2^ (Figure 6e). The lap−shear tests (Figure S27) indicated that the adhesion toughness of MnO_2_/CNTs−T−PAC (68.0 kPa) and AC/CNTs−T−PCC (88.6 kPa) made by AIP method was more than one time stronger than those made via DS approach (33.9 and 43.6 kPa) (Figure 6f).

The asymmetric SC (Figure 6g) assembled by AIP+ISP approach showed an extended voltage window of 1.8 V (Figure 6h), excellent capacitive behavior, and rate performance (Figure S28). The specific capacitance of the asymmetric SC by AIP+ISP was enhanced to 199.4 mF cm^−2^ (responding to an energy density of 84.2 µWh cm^−2^) at the current density of 0.7 mA cm^−2^. For comparison (Figure 6i,j), the SC fabricated through DS showed a relatively low specific capacitance of 51.1 mF cm^−2^. The high performance of AIP+ISP−built asymmetric SC can be attributed to its low series resistance and rapid surface ions diffusion (Figure 6k), which was provided by the compact seamless electrode/electrolyte interface. The asymmetric SC possessed a self−discharge time (Figure 6l) as long as 123 716 s (34.4 h from 1.8 to 0.5 V), and it can remain 90.4% of its initial capacitance after 20 000 charge/discharge cycles (Figure 6m), due to its excellent structural stability (Figure S29). Figure 6n showed two asymmetric SCs in series to power a flower‐shaped diode (working voltage of 3 V) under flat and bending (180°) states.

## 3 Conclusions

In summary, we proposed a universal strategy to achieve ultrastable flexible supercapacitors with ultralong cyclic stability by constructing multiple seamless interlocked interfaces. The tough electrode/electrolyte interface connected by covalent bonds was constructed by an anchored interfacial polymerization. Through this design, the interfacial toughness between polymer electrolyte and electrode was boosted by over two orders, and interfacial adhesion was also enhanced by one order, compared with the traditional direct stacking approach. Meanwhile, a bilayer heterogeneous polycation/polyanion electrolyte with an interlocked interfacial structure was constructed by in situ polymerization, which can efficiently regulate the self−discharge of the built supercapacitor through the strong electrostatic interactions between the charged polyelectrolyte and ion accumulated on/in electrode. As desired, the resultant supercapacitors with multiple seamless interfaces not only exhibited superior electrochemical performance (high capacitance, slow self−discharge rate, and long cyclic lifetime), but also possessed ultrahigh flexibility even after being folded for 100 000 times. More importantly, this approach is available to build flexible supercapacitors based on both electric double−layer and pseudocapacitive electrode materials, and the demonstrated interfacial strengthening strategy can also be extended to develop other flexible electronics with enhanced structural stability.

## Conflicts of Interest

The authors declare no conflicts of interest.

## Supporting information




**Supporting File**: advs75418‐sup‐0001‐SuppMat.docx.

## Data Availability

The data that support the findings of this study are available in the supplementary material of this article.
