# Supplementary material for: Constructing Seamless Interfaces for Ultrastable Flexible Supercapacitors
Source: Adv Sci (Weinh). 2026 Apr 21;13(40):e75418. doi: 10.1002/advs.75418 (PMC13335712; doi:10.1002/advs.75418)
Supplement: Supplementary file 1 — Supporting File: advs75418‐sup‐0001‐SuppMat.docx. [file ADVS-13-e75418-s001.docx]

**Supporting Information**

**Constructing seamless interfaces for ultrastable flexible supercapacitors**

Keyi Dong, Zefeng Yan, Weiyang Tang, Quanhu Sun, Jiaxin Yang, Yan Tan, Yu Wang, Tian Lv, Tao Chen*

Shanghai Key Lab of Chemical Assessment and Sustainability, School of Chemical Science and Engineering, Tongji University, Shanghai 200092, P. R. China. e-mail: tchen@tongji.edu.cn

**Experimental Procedures**

**Chemical modification of CNTs film.**

CNTs film (thickness was 10 μm) was soaked in concentrated nitric acid for 12 h, followed by rinsing with deionized water to get carboxylated CNTs (CNTs−COOH) film. Then, the CNTs−COOH film was soaked in an ethanol solution containing TMSPMA (2.0 wt%) and water (3.0 wt%) for 48 h at room temperature, the obtained silane−grafted CNTs (CNTs−COO−TMSPMA) film was rinsed with deionized water and dried overnight.

**Fabrication of supercapacitor through anchored interfacial polymerization and in−situ polymerization (AIP+ISP) method.**

The DEAP (100 μL) was added to ethyl acetate (5 mL) to obtain an initiator solution, which (10 μL) was dropped on the surface of the CNTs−COO−TMSPMA film placed on a glass slide. After the ethyl acetate was evaporated, a home−made silicone mold with 800 μm depth hole was placed on the CNTs−COO−TMSPMA film. The pre−gel solution of PAC was composed of acrylamide (8 M), acrylic acid (2 M), 3−sulfopropyl methacrylate potassium salt (SPMA, 2 M) and methylene−bis−acrylamide (MBA, 0.1 wt%). The pre−gel solution of PAC was poured into the mold, and covered with another glass slide. The sample were placed under a UV lamp (365 nm, 300 W) for 30 min to obtain CNT−COO−TMSPMA film grafted with PAC electrolyte. CNT−COO−TMSPMA film grafted with PCC electrolyte was prepared with similar process, with using a pre−gel solution of PCC containing acrylamide (8 M), acrylic acid (2 M), methacryloxyethyltrimethyl ammonium chloride (MATAC, 2 M), and MBA (0.1 wt%). The pre−gel solution of PCC was added to another CNT−COO−TMSPMA film coated with DEAP, then the previously prepared CNT−COO−TMSPMA film grafted with PAC electrolyte was put on it. After UV irradiation for 30 min, the supercapacitor with multiple seamless interlocked interfaces was obtained.

**Fabrication of supercapacitor through anchored interfacial polymerization and direct staking (AIP+DS) method.**

The supercapacitor was assembled by directly pressing a piece of CNT−COO−TMSPMA film grafted with PAC electrolyte and a piece of CNT−COO−TMSPMA film grafted with PCC electrolyte together.

**Preparation of supercapacitor through direct staking and in−situ polymerization (DS+ISP) method.**

Initiator of 2−hydroxy−4'−(2−hydroxyethoxy)−2−methylpropiophenone (I2959, 0.2 wt%) was add to PAC and PCC pre−gel solution, respectively. The PAC pre−gel solution with initiator was transferred into an 800 μm depth home−made silicone mold, and followed by free radical polymerization under a UV lamp for 30 min. Then, the PCC pre−gel solution was poured into another mold with the same depth placed on the polymerized PAC electrolyte, followed by polymerization under a UV lamp for 30 min to obtain PAC/PCC electrolyte. The supercapacitor was assembled by sandwiching a piece of PAC/PCC electrolyte with two pieces of CNT−COO−TMSPMA film electrodes.

**Preparation of supercapacitor through all direct staking (DS) method.**

The PAC and PCC electrolyte films were individually fabricated by the same method above. The supercapacitor was assembled by sandwiching two pieces of PAA and PCC electrolyte films with two pieces of CNT−COO−TMSPMA film by directly layer−by−layer stacking process.

**Preparation of supercapacitor through** **conventional in−situ polymerization.**

A home−made silicone mold with 800 μm depth hole was placed on a piece of CNTs film on a piece of glass slide. The pre−gel solution of PAC with initiator (I2959, 0.2 wt%) was poured into the mold, and covered with another piece of glass slide. The sample was placed under a UV lamp (365 nm, 300 W) for 30 min to obtain CNTs film with PAC electrolyte. The pre−gel solution of PCC with initiator (I2959, 0.2 wt%) was added to another CNTs film, then the previously prepared CNTs film with PAC electrolyte was put on it. After UV irradiation for 30 min, the supercapacitor was obtained.

**Fabrication and surface modification of AC/CNTs film.**

AC powder, carbon black (Super P) and polyvinylidene fluoride (PVDF) binder were dispersed in a N−methyl−2−pyrrolidone (NMP) with a mass ratio of 8:1:1, and was stirred slightly for 6 h to form a homogeneous slurry. The slurry was blade−coated on CNTs film, and followed by drying at 80 ^o^C for 12 h to fabricate AC/CNTs film (mass loading was 3.37 mg cm^−2^). After that, the films were treated with O_2_ plasma for 5 min, and then soaked in an ethanol solution containing TMSPMA (2.0 wt%) and water (3.0 wt%) for 48 h at room temperature, achieving silane−grafted AC/CNTs (AC/CNTs−T) film after rinsed with deionized water and dried overnight.

**Fabrication and surface modification of MnO_2_/CNTs film.**

The MnO_2_/CNT film were synthesized through an electrochemical method in a three−electrode system, where the CNTs film, a platinum sheet, and Ag/AgCl were used as the working electrode, counter electrode, and reference electrode, respectively. The mixed aqueous solution containing MnSO_4_ (0.05 M), CH_3_COONa (0.05 M) and ethanol (10 vol%) was used as the electrolyte. The electrochemical deposition was conducted at a constant current density of 5.0 mA cm^−2^ for 35 minutes. The as−prepared MnO_2_/CNTs film (mass loading was 3.30 mg cm^−2^) was rinsed with deionized water several times, followed by drying at 60 °C for 2 h. After that, the film was soaked in an ethanol solution containing TMSPMA (2.0 wt%) and water (3.0 wt%) for 48 h at room temperature, rinsed with deionized water and dried overnight to get silane−grafted MnO_2_/CNTs (MnO_2_/CNTs−T) film.

**Preparation of asymmetric supercapacitors through AIP+ISP, AIP+DS, DS+ISP and DS method.**

The preparation method was similar to the supercapacitors with CNTs−COO−TMSPMA film as electrodes. It should be noted that MnO_2_/CNTs−T film was coated with PAC while AC/CNTs−T film was coated with PCC.

**Characterization and calculations.**

The morphologies of electrodes and electrolytes were characterized by field scanning electron microscopy (FESEM, Hitachi S−4800). The electrochemical performance of supercapacitors was measured by an electrochemical workstation (CHI 760E, Shanghai Chenhua). The contact angle (CA) measurements were performed using a contact angle meter (JY−82C, Chengde Dingsheng). The chemical species were analyzed by an X−ray photoelectron spectroscopy (XPS, Thermo Scientific K−Alpha). The interfacial toughness was measured by 180^o^ peeling test using a mechanical test machine (HY−0350, Shanghai Hengyi Co. Ltd). The adhesion test was conducted by a mechanical test machine (HY−0350, Shanghai Hengyi Co. Ltd) with a standard lap−shear method. The supercapacitors were encapsulated in commonly used plastic film to prevent loss of H_2_O from the hydrogel electrolytes during long-term measurements.

The ion diffusion coefficients (D) of polymer electrolyte in CNTs-based SCs were calculated according to galvanostatic intermittent titration technique (GITT) curves by the following equation (W. Weppner and R. A. Huggins. *J. Electrochem. Soc.*, **1977**,

*124*, 1570):

$$D=\frac{4}{\pi\tau}(\frac{m_{B}V_{m}}{M_{B}S})(\frac{{\Delta E}_{s}}{{\Delta E}_{t}})$$

where *τ* is the pulse duration, *m_B_* represents the mass of CNTs, *V_m_* represents the molar volume of CNTs, *M_B_* represents the molar mass of CNTs, *S* is the interfacial area, and *ΔE_s_* and *ΔE_t_* are the steady-state voltage change and transient voltage change, respectively.

**Simulation details.**

The interaction energy between CNTs−COO−TMSPMA and a unit of PAM after DS as well as the dissociation energy of the covalent bond after AIP was obtained by density functional theory (DFT) calculation. All DFT calculations were performed using DMol3 module with generalized gradient approximation (GGA) and Perdew−Burke−Ernzerhof functional in BIOVIA Material Studio (MS) 2019. The interaction energy (E_inter_) was calculated according to the following formula:

$$E_{inter}=E_{total}-E_{CNTs-COO-TMSPMA}-E_{PAM}$$

The dissociation energy (E_disso_) was calculated according to the following formula:

$$E_{disso}=E_{total}-E_{CNTs-COO-TMSPMA\cdot}-E_{PAM\cdot}$$

Where the spin multiplicity was set as doublet.

The stress distribution of the supercapacitors with different interfaces under shear stress was simulated using solid mechanics module by COMSOL Multiphysics 6.2. The simulation was mainly about the horizontal relative motion of two planes in shear mode. The hexahedral mesh improved the accuracy of the mesh and avoided the difficulty of convergence caused by irregular deformation.


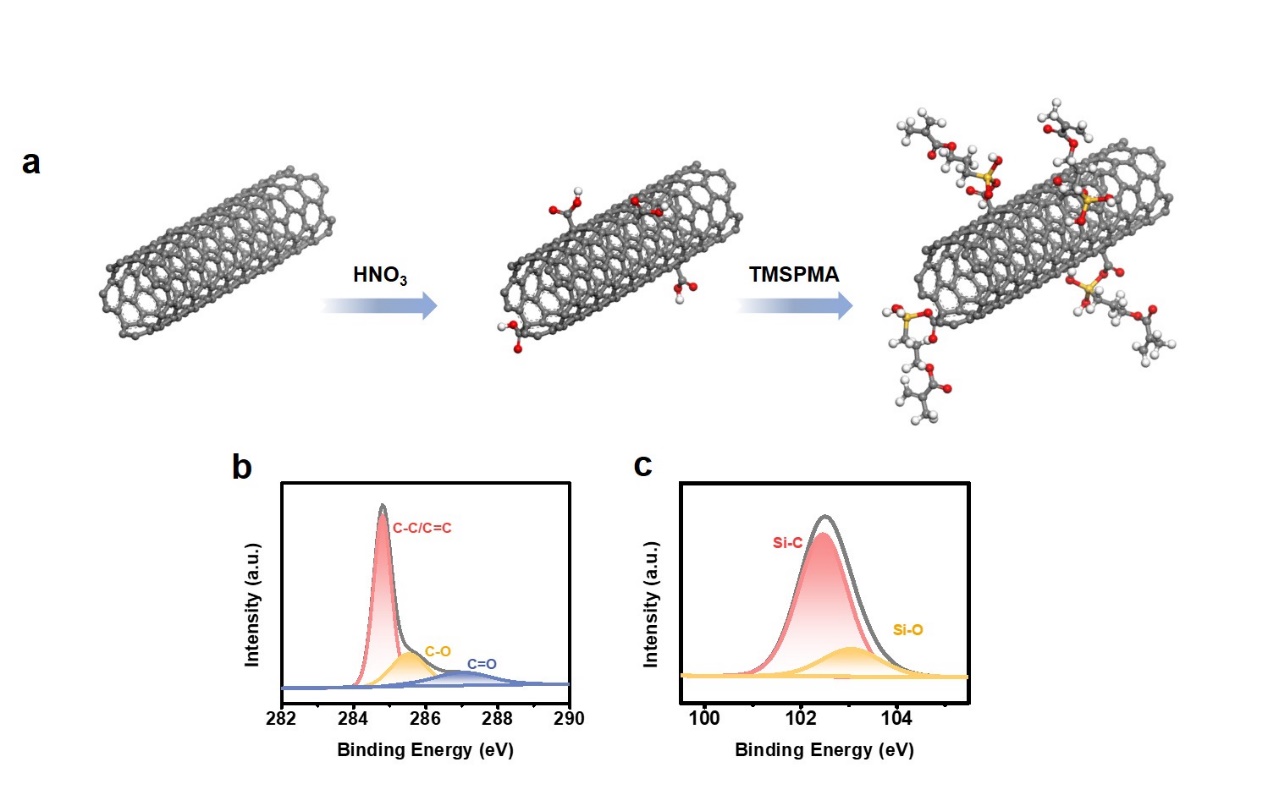


**Figure S1.** a) Surface chemical modification of carbon nanotube. The XPS spectra of b) C 1s and c) Si 2p of the surface groups of CNTs−COO−TMSPMA film.


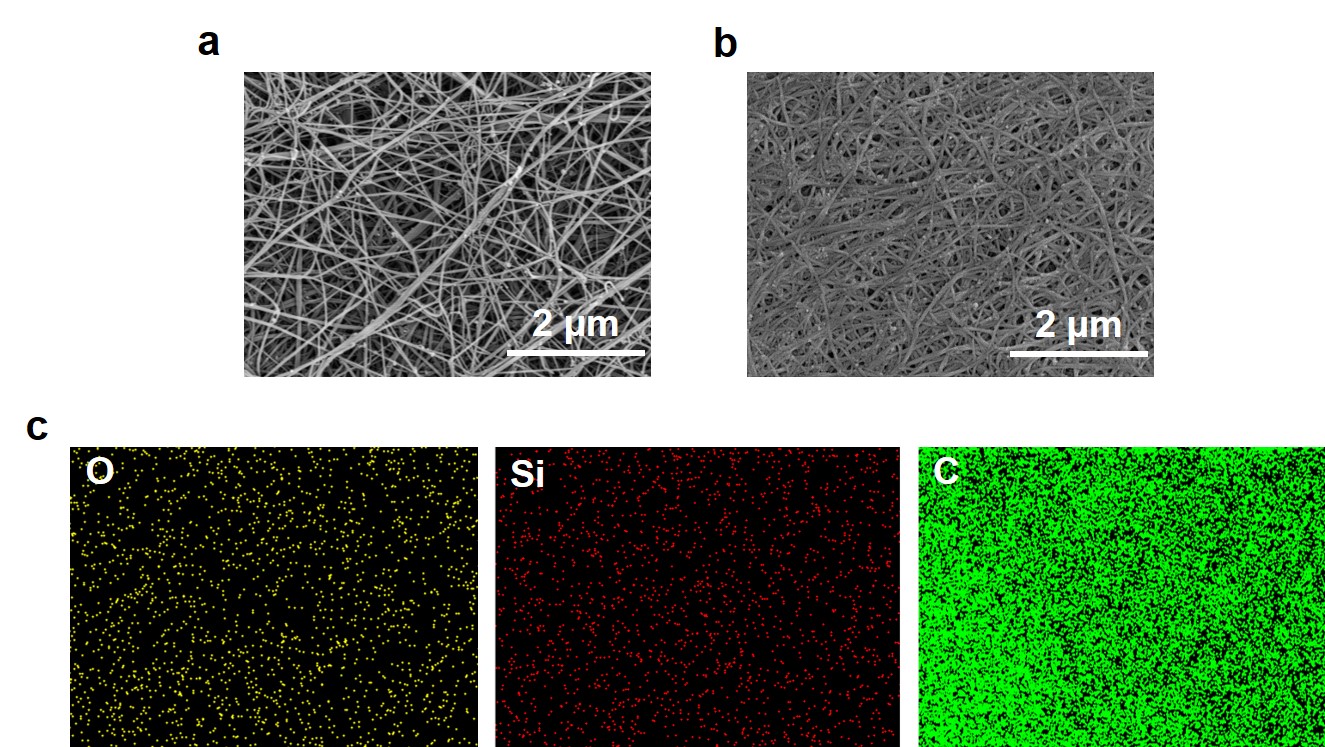


**Figure S2.** SEM image of CNTs films a) before and b) after modification. c) The energy dispersive spectroscopy (EDS) mapping of CNTs−COO−TMSPMA film.


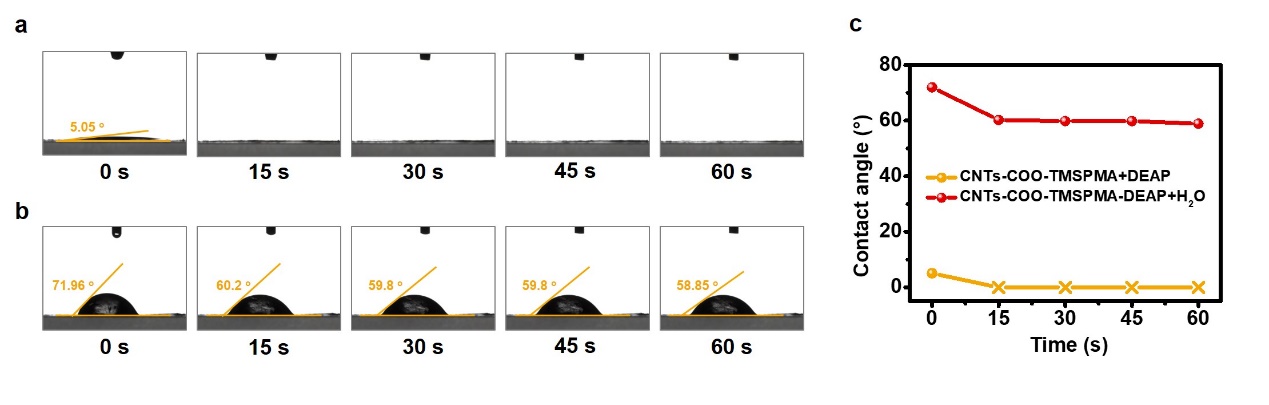


**Figure S3.** a) Contact angle of CNT−COO−TMSPMA film to DEAP dissolved with initiators. b) Water contact angle of CNT−COO−TMSPMA film with initiator layer. c) The excellent wettability of CNTs−COO−TMSPMA film to DEAP dissolved in ethyl acetate and subsequent hydrophobicity of the film to water.


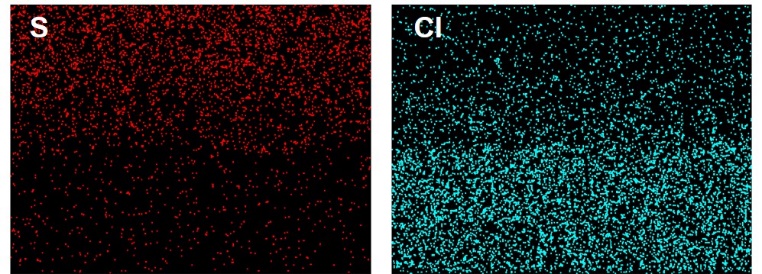


**Figure S4.** The EDS mapping of bilayer PAC−PCC polymer electrolyte via ISP.


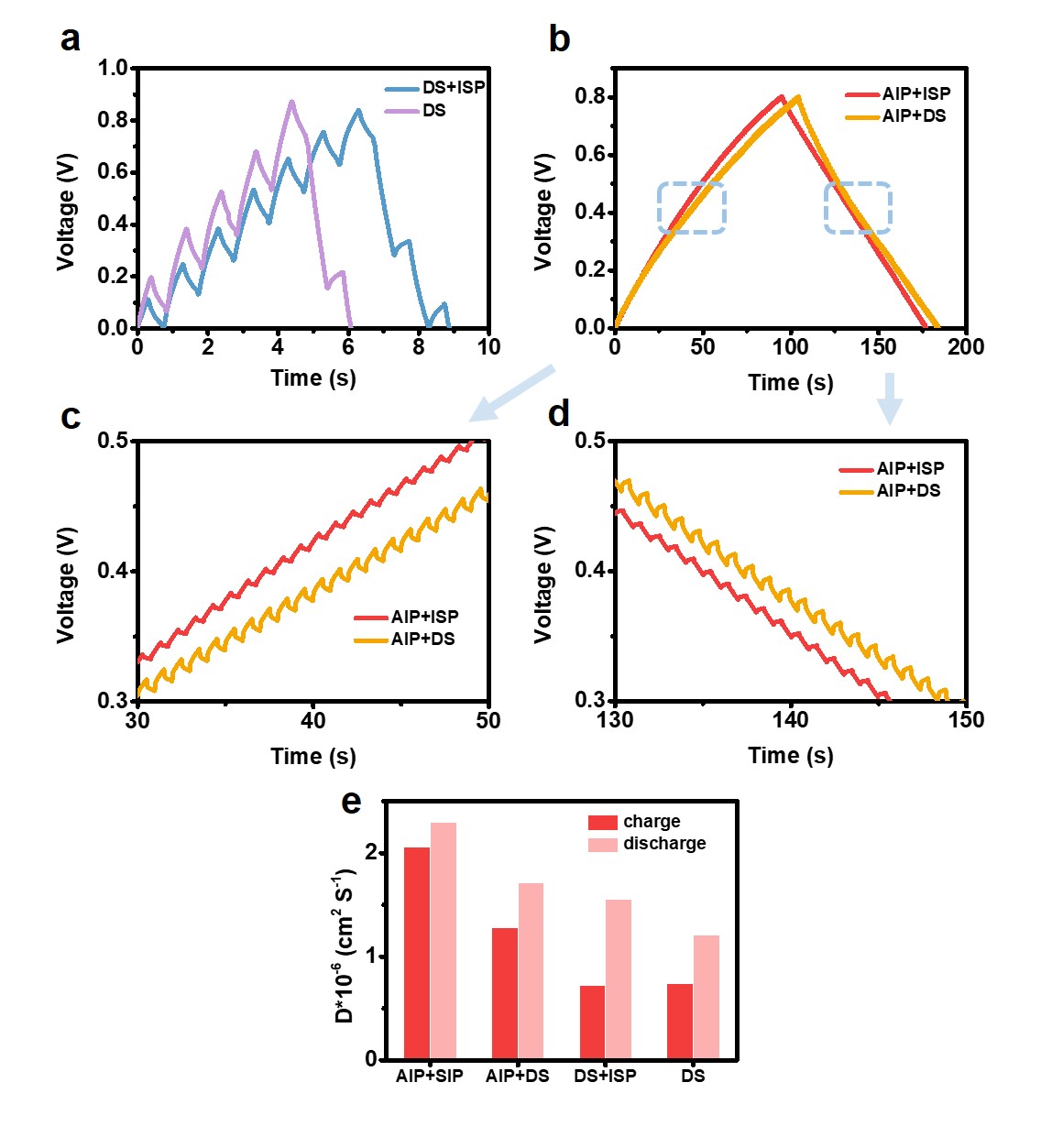


**Figure S5.** a−d) GITT curves of CNTs-based SCs fabricated by different methods. e) Ion diffusion coefficients of polymer electrolytes in SCs during charge or discharge process.


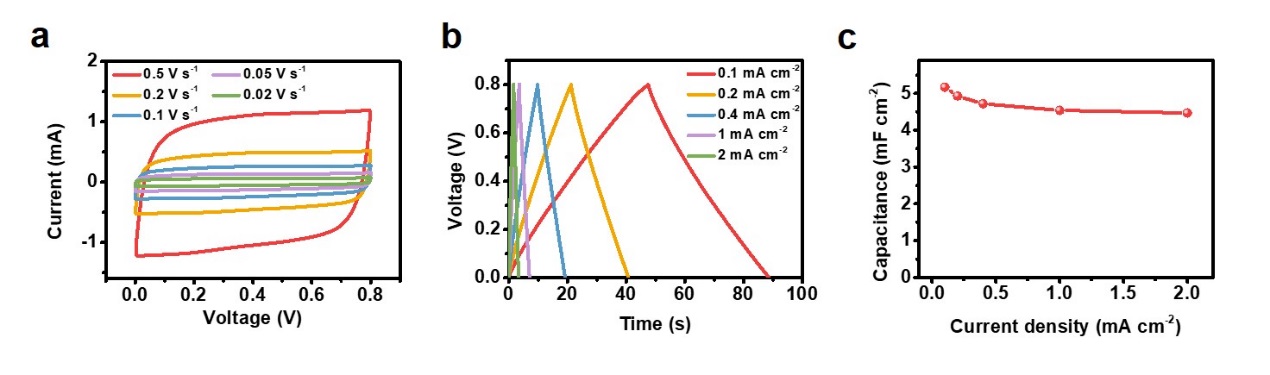


**Figure S6.** Electrochemical performance of supercapacitor based on AIP+ISP by using CNTs films as electrodes. a) CV curves at different scanning rates. b) GCD curves at different current densities. c) Dependance of areal specific capacitance on the current densities.


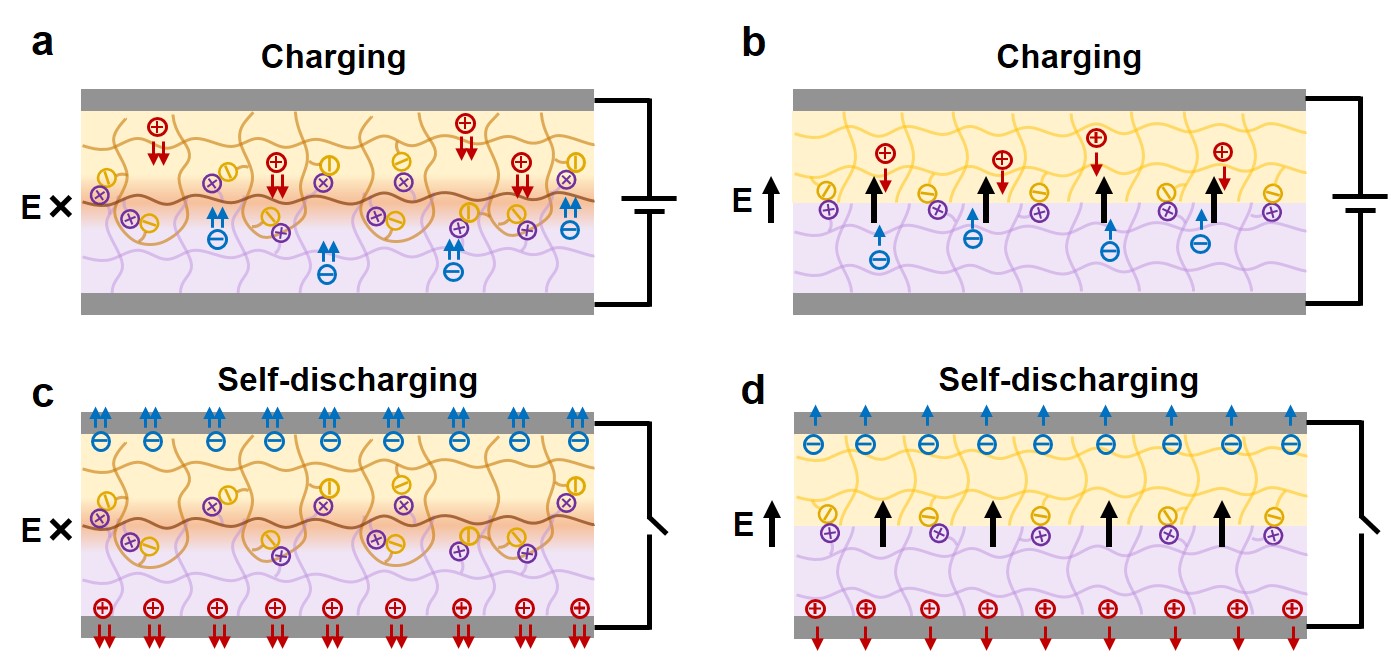


**Figure S7.** Schematic diagrams to show a), b) charging and c), d) self−discharging processes of SCs assembled through ISP and DS methods.


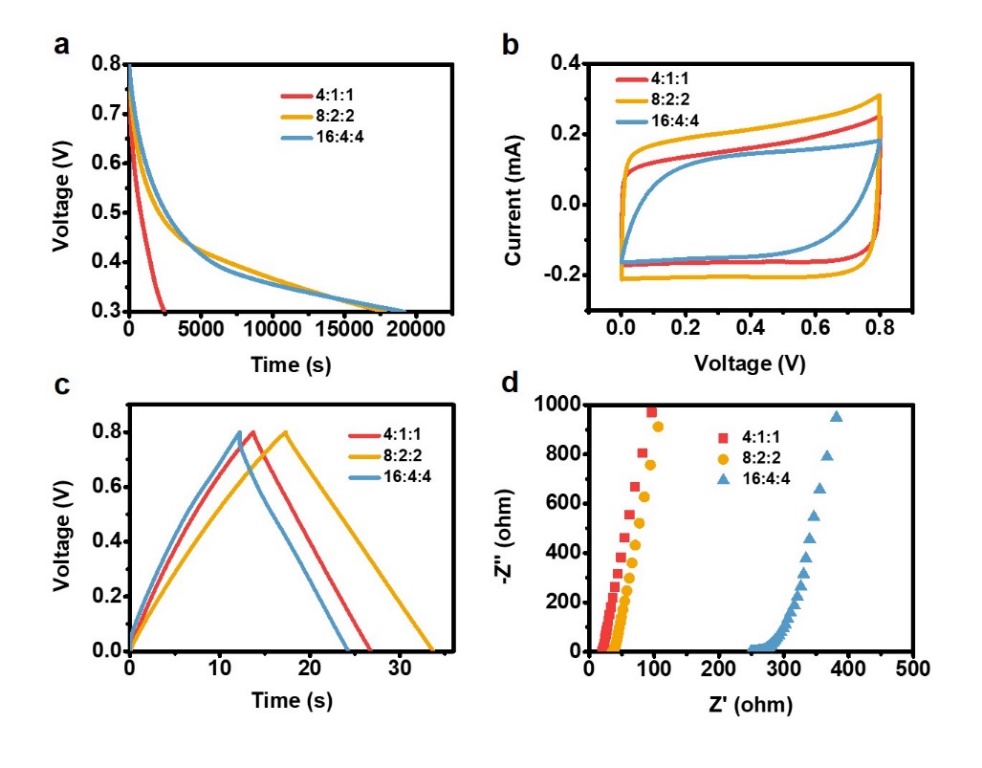


**Figure S8.** Electrochemical performance of SCs with different monomer concentrations (AM: AA: MATAC/SPMA). a) Self−discharge curves from 0.8 V to 0.3 V. b) CV curves at a scan rate of 100 mV s^-1^. c) GCD curves at a current density of 0.2 mA cm^-2^. d) Nyquist plots of the SCs.


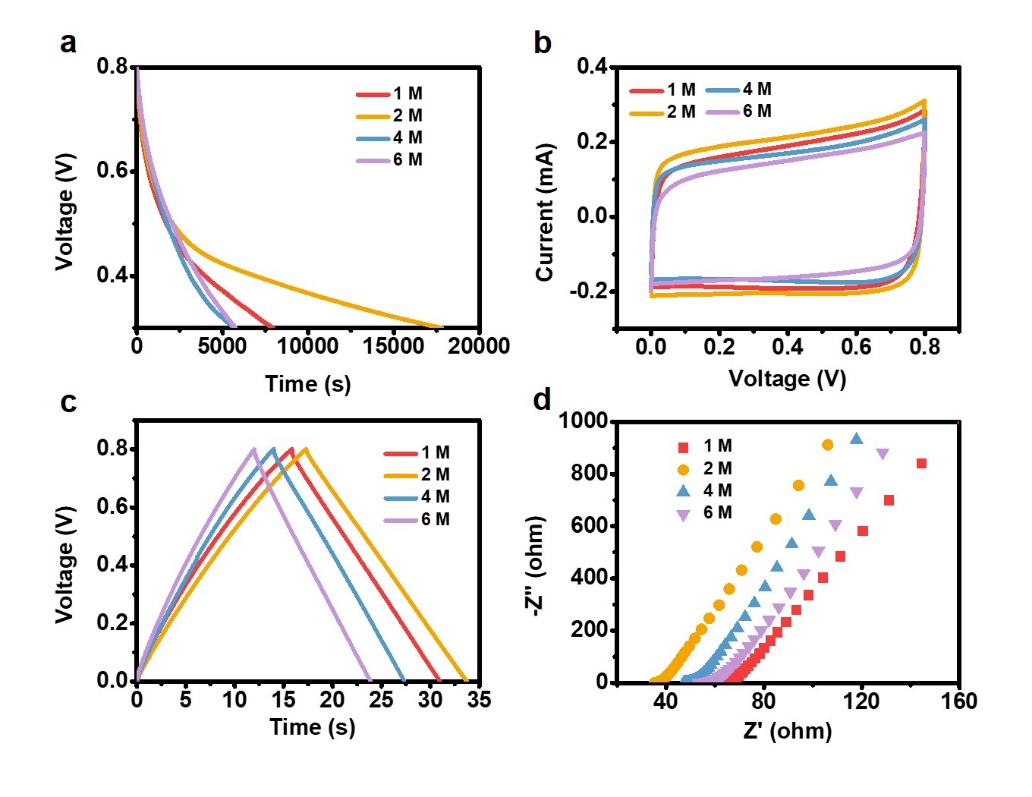


**Figure S9.** Electrochemical performance of SCs with different anionic/cationic monomer concentrations (AM: AA=8:2). a) Self-discharge curves from 0.8 V to 0.3 V. b) CV curves at a scan rate of 100 mV s^-1^. c) GCD curves at a current density of 0.2 mA cm^-2^. d) Nyquist plots of the SCs.


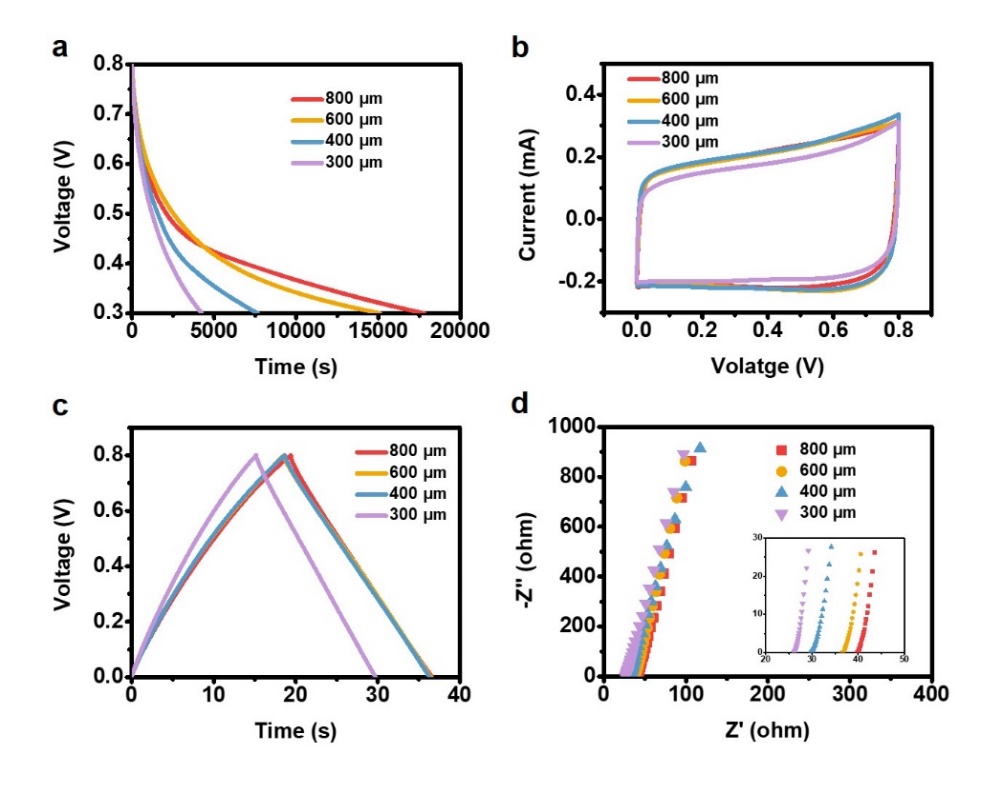


**Figure S10.** Electrochemical performance of SCs with different thickness of anionic/cationic hydrogel. a) Self−discharge curves from 0.8 V to 0.3 V. b) CV curves at a scan rate of 100 mV s^-1^. c) GCD curves at a current density of 0.2 mA cm^-2^.(d) Nyquist plots of the SCs.

**
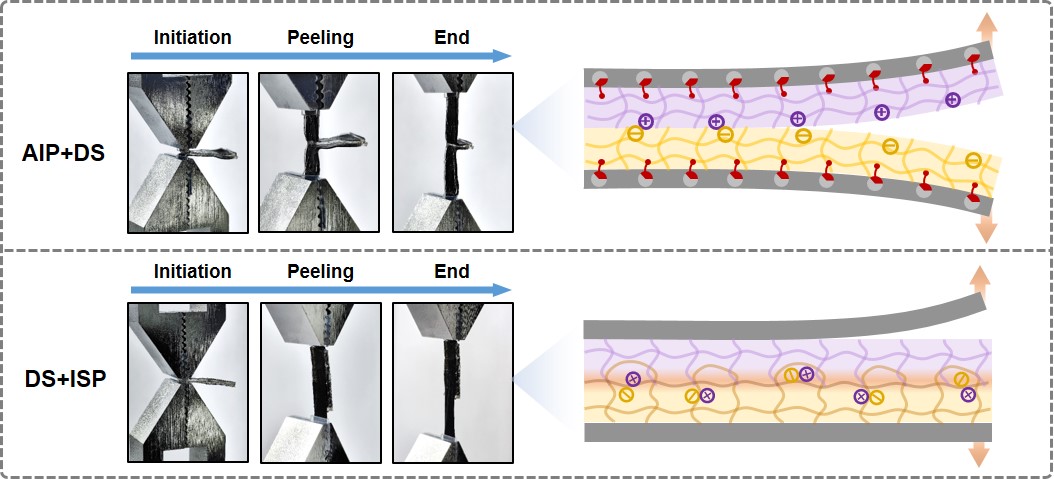
**

**Figure S11.** Optical images and schematic diagrams of the 180 ^o^ peeling tests for SCs assembled by AIP+DS and DS+ISP.


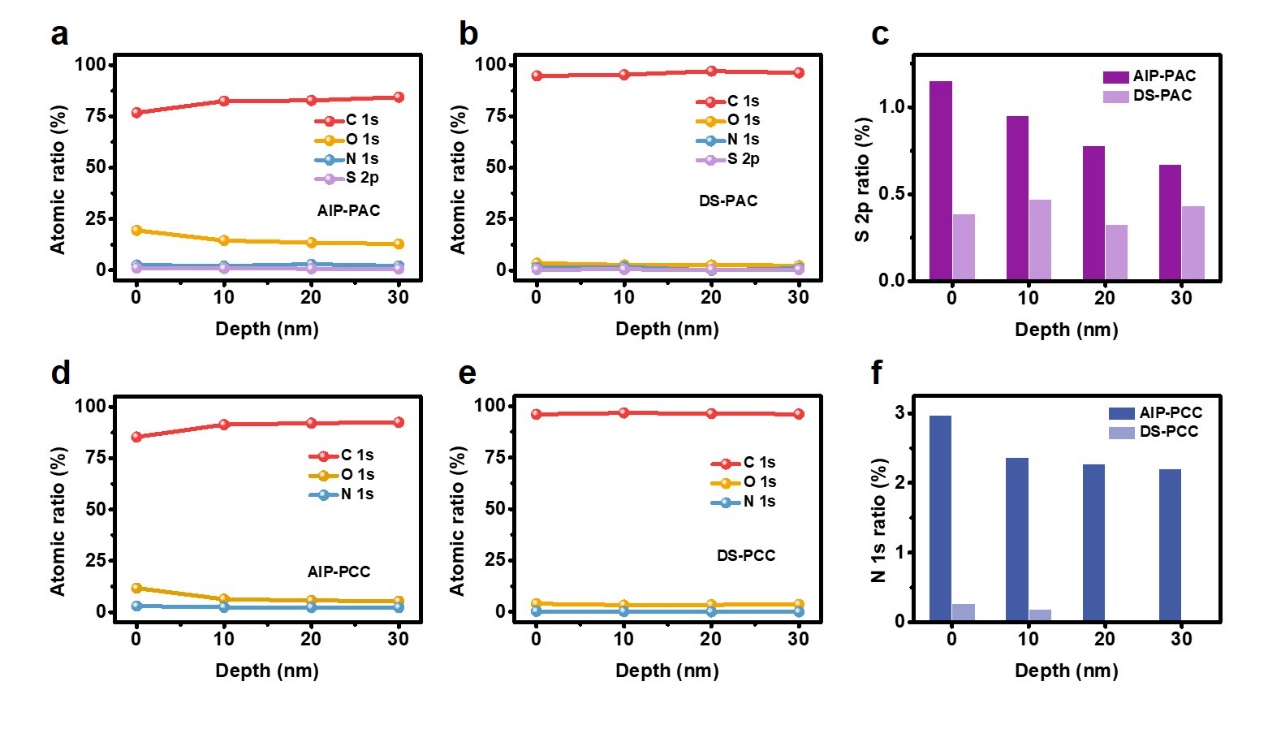


**Figure S12.** XPS depth profiling of the peeled electrodes with PAC fabricated by AIP (a) and DS (b). c) S 2p ratio versus etching depth of (a) and (b) in details. XPS depth profiling of the peeled electrodes with PCC prepared by AIP (d) and DS (e). f) N 1s ratio versus etching depth of (d) and (e) in details.


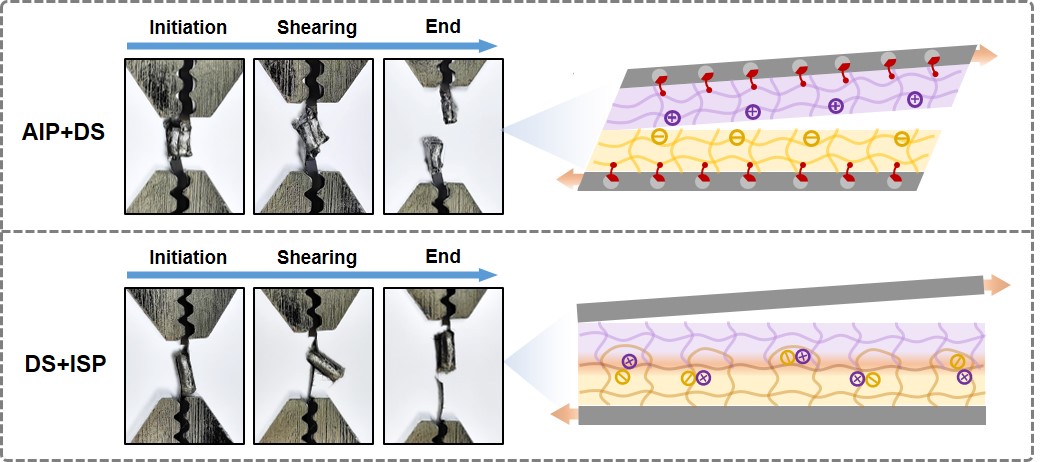


**Figure S13.** Optical images and schematic diagrams of the lap-shear tests for SCs assembled by AIP+DS and DS+ISP.


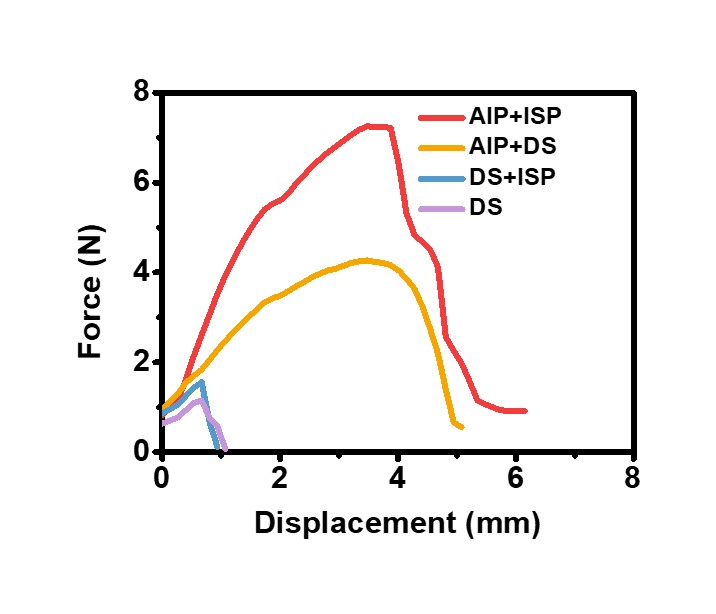


**Figure S14.** Lap−shear force−displacement curves of SCs with different contact interfaces.


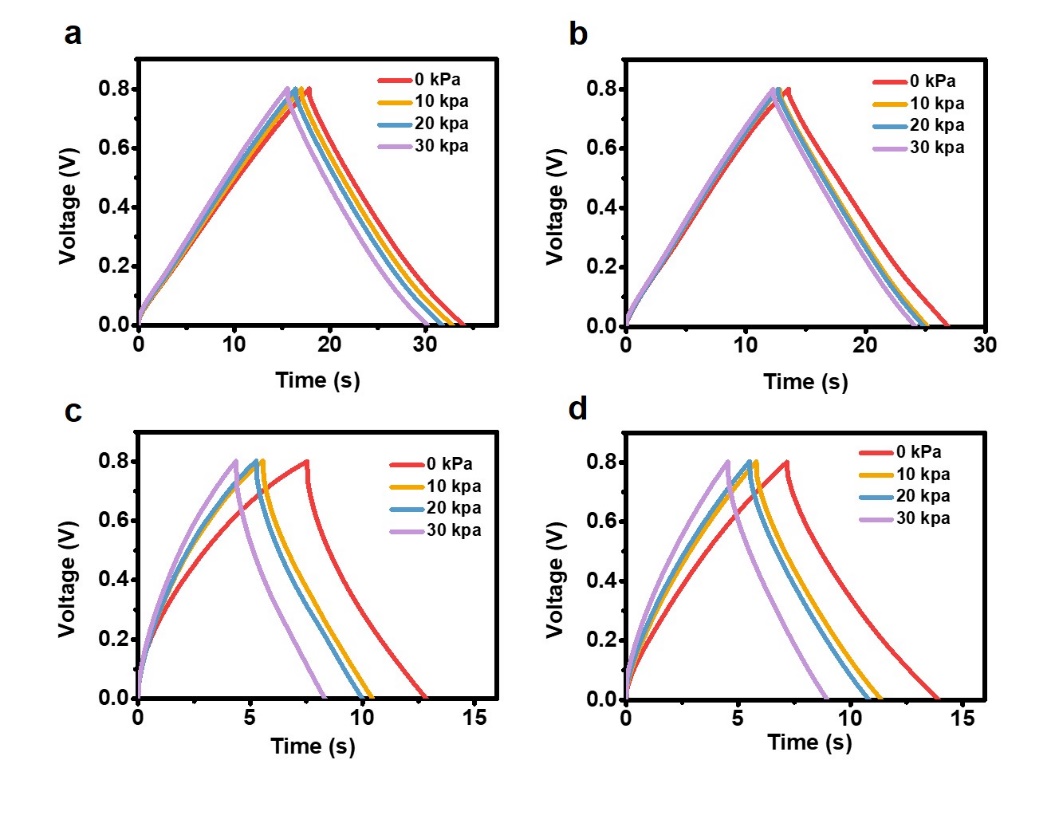


**Figure S15.** GCD curves of SCs assembled by a) AIP+ISP, b) AIP+DS, c) DS+ISP and d) DS assembling approaches under various shear stresses.


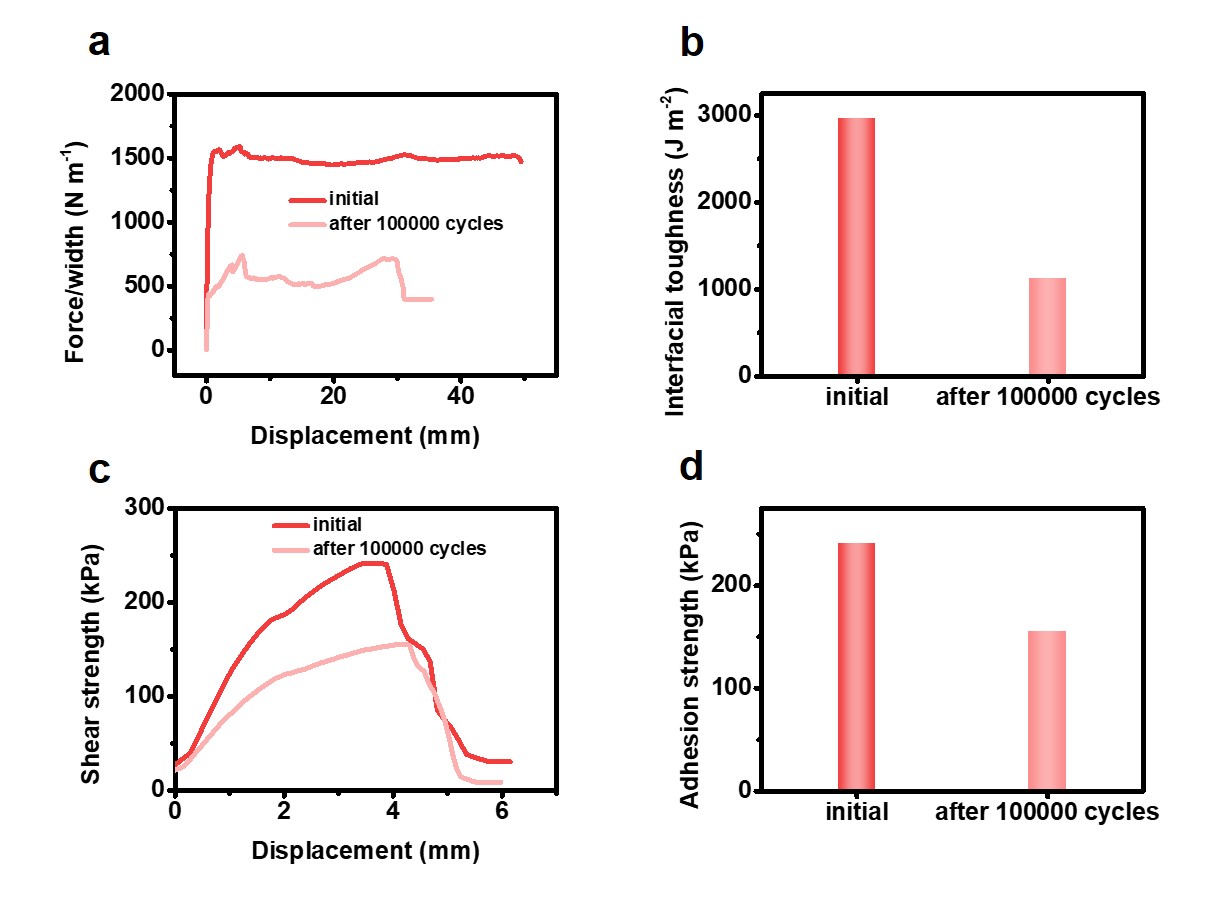


**Figure S16.** a) The 180° peeling force per width versus displacement curves of the SCs fabricated by AIP+ISP before and after 100,000 charge/discharge cycles. b) Interfacial toughness between electrode and electrolyte calculated from (a). c) Lap−shear force−displacement curves of the SCs fabricated by AIP+ISP before and after 100,000 charge/discharge cycles. d) Adhesion strength between electrode and electrolyte calculated from (c).

*
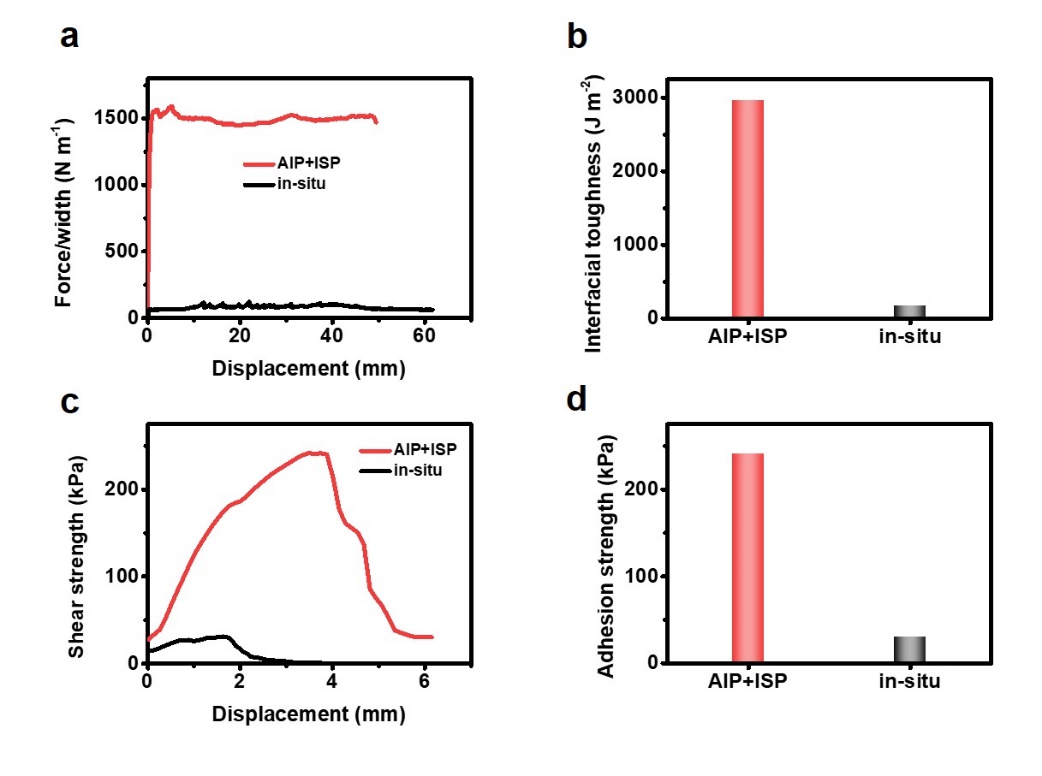
*

**Figure S17.** a) The 180 ° peeling force per width versus displacement curves of the SCs fabricated by AIP+ISP and in−situ polymerization. b) Interfacial toughness between electrode and electrolyte calculated from (a). c) Lap−shear force−displacement curves of the SCs fabricated by AIP+ISP and in−situ polymerization. d) Adhesion strength between electrode and electrolyte calculated from (c).


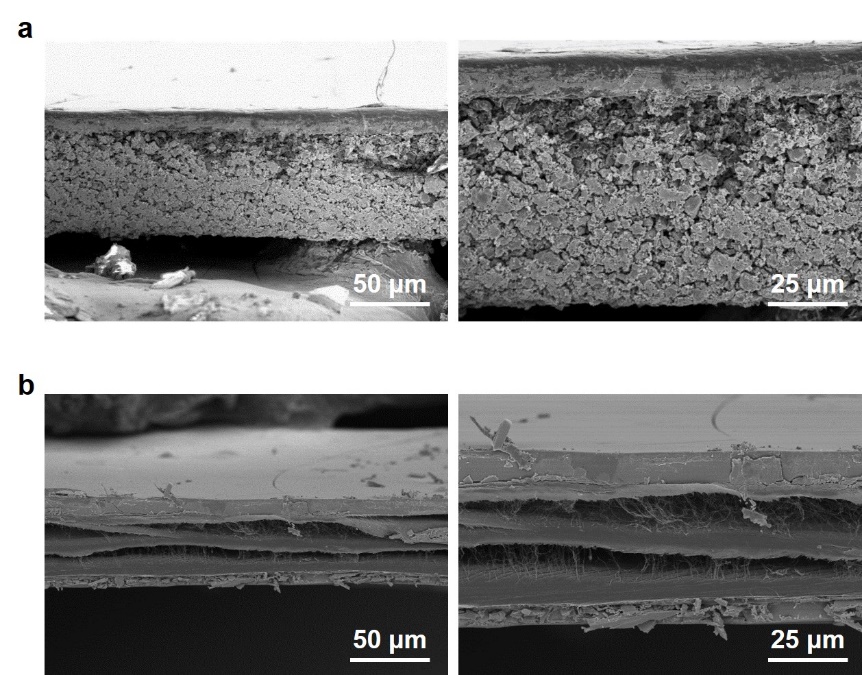


**Figure S18.** Cross−sectional SEM images of a) AC/CNTs and b) MnO_2_/CNTs films.


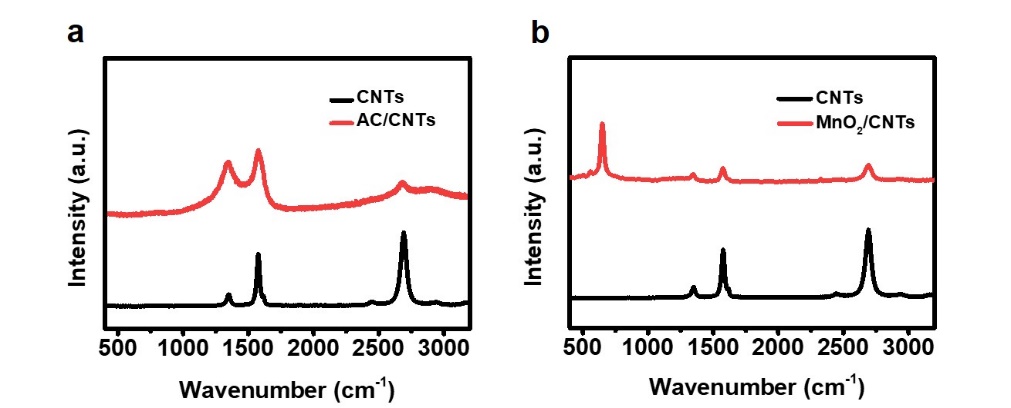


**Figure S19.** Raman spectra of a) AC/CNTs and b) MnO_2_/CNTs films.


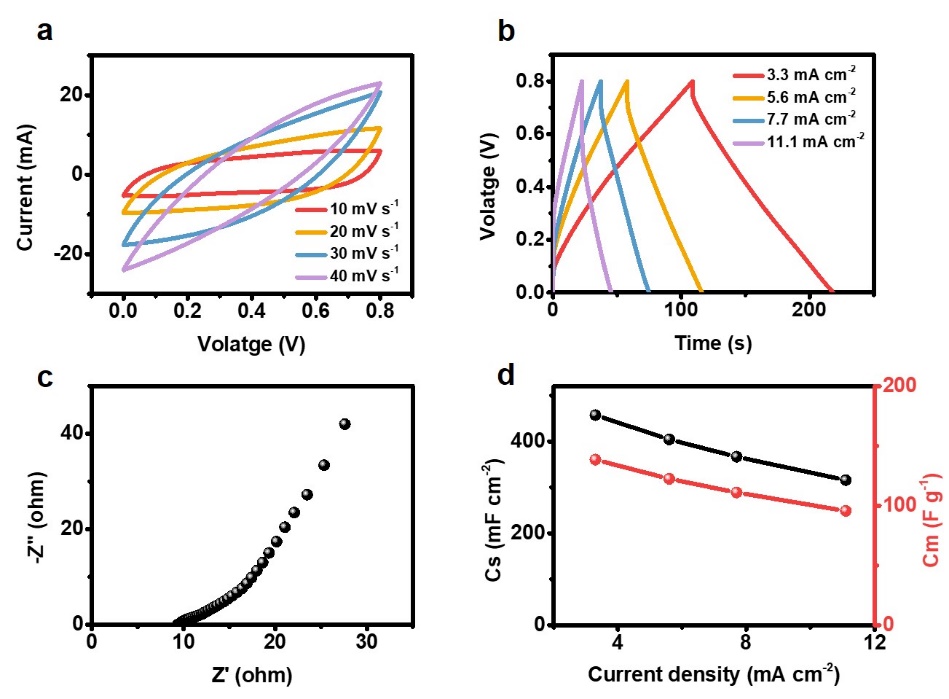


**Figure S20.** Electrochemical performance of MnO_2_/CNTs electrodes measured in a three−electrode system. a) CV curves at different scanning rates. b) GCD curves at different current densities. c) Nyquist plots of the SC. d) Areal specific capacitance and mass specific capacitance as functions of current densities.


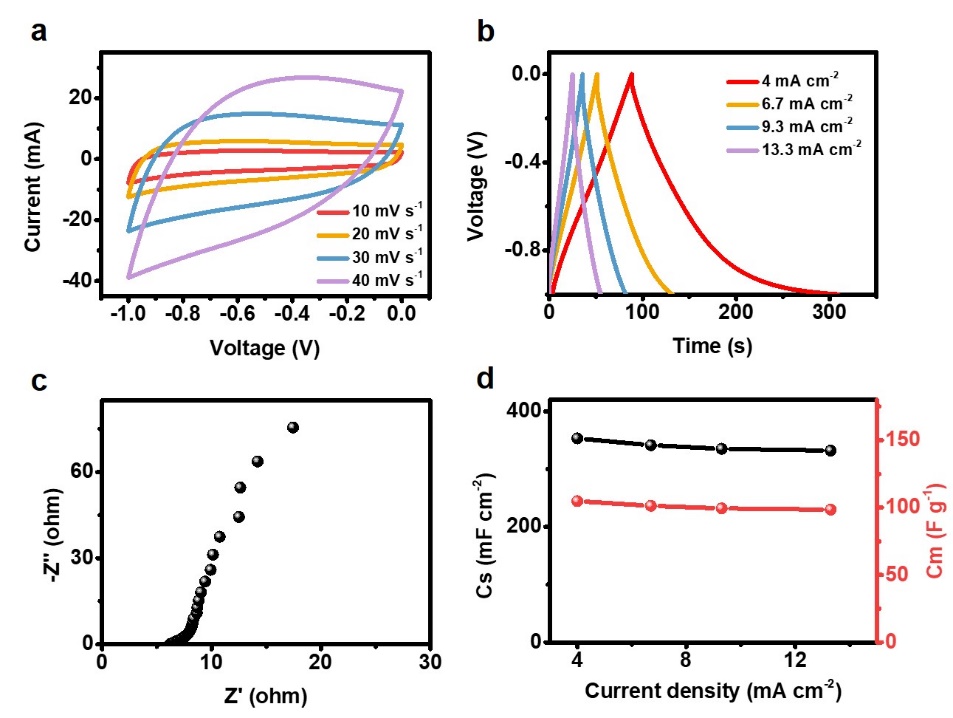


**Figure S21.** Electrochemical performance of AC/CNTs electrodes measured in a three−electrode system. a) CV curves at different scanning rates. b) GCD curves at different current densities. c) Nyquist plots of the SC. d) Areal specific capacitance and mass specific capacitance as functions of current densities.


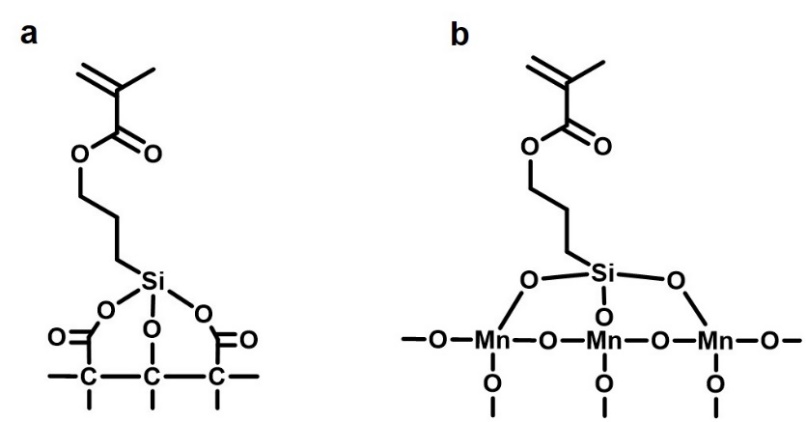


**Figure S22.** Schematic to show the surface chemical modification on a) AC/CNTs electrodes and b) MnO_2_/CNTs electrodes.


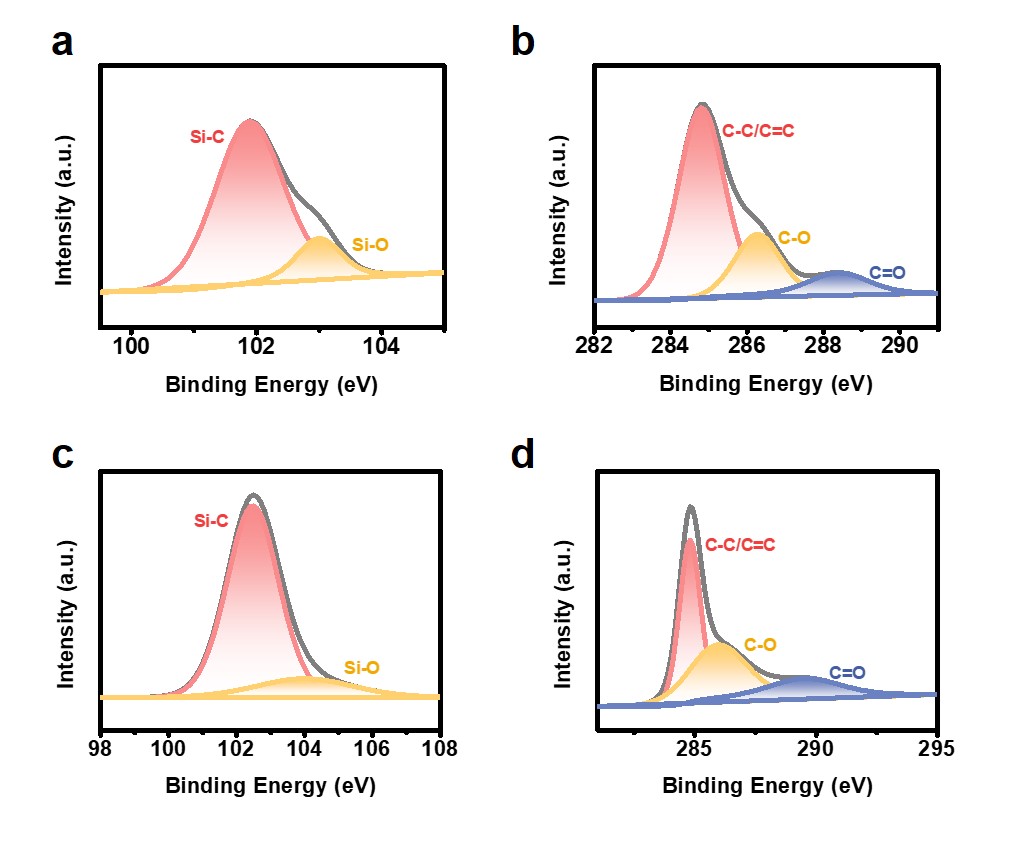


**Figure S23.** The XPS spectra of a) Si and b) C of the surface groups of MnO_2_/CNTs−T electrodes. The XPS spectra of c) Si and d) C of the surface groups of AC/CNTs−T electrodes.


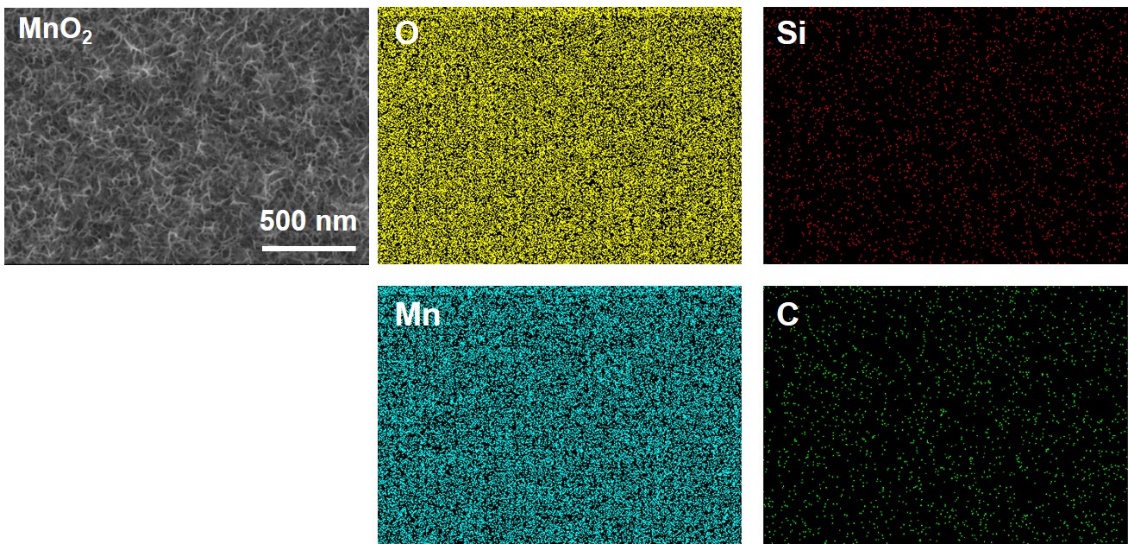


**Figure S24.** The EDS mapping of MnO_2_/CNTs−T.


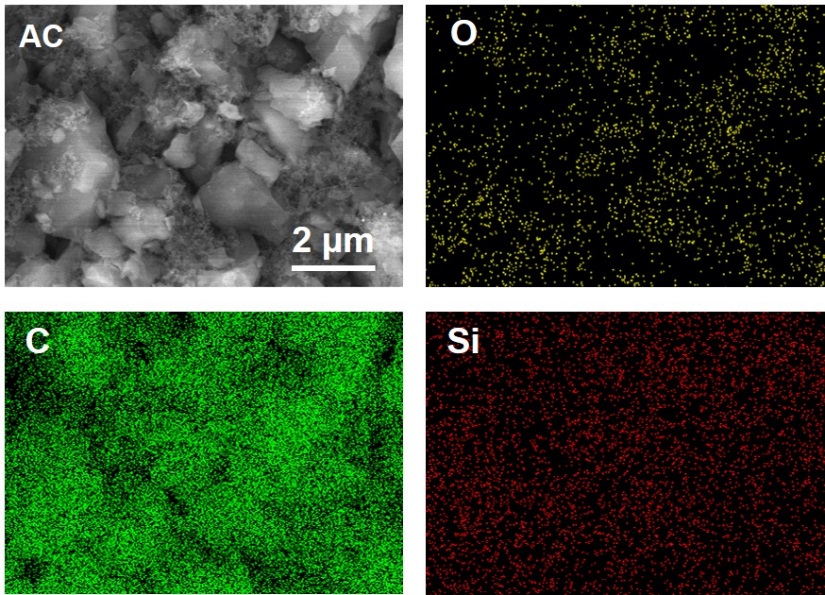


**Figure S25.** The EDS mapping of AC/CNTs−T.


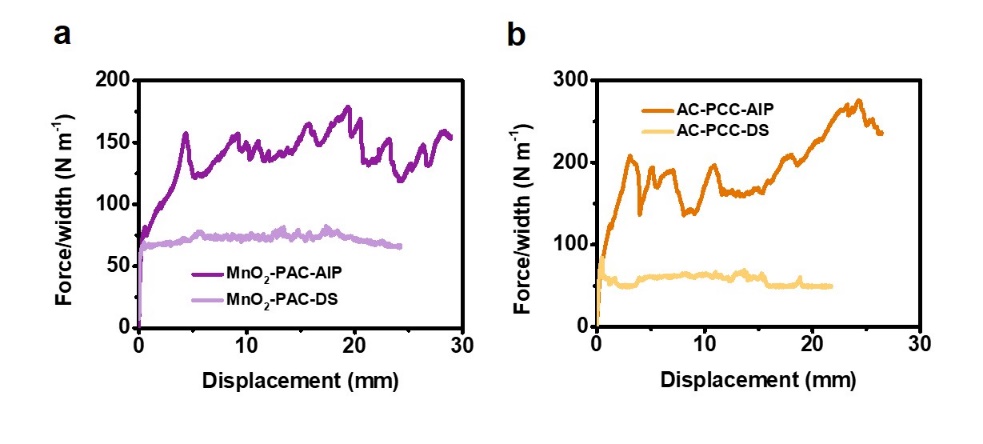


**Figure S26.** The 180 ° peeling force−displacement curves of a) PAC on MnO_2_/CNTs−T and b) PCC on AC/CNTs−T via AIP and DS, respectively.


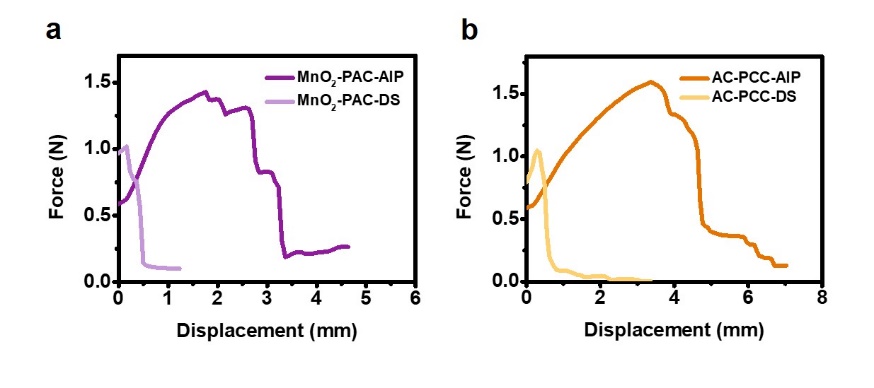


**Figure S27.** Lap−shear force−displacement curves of a) PAC to MnO_2_/CNTs−T and b) PCC to AC/CNTs−T via AIP and DS, respectively.


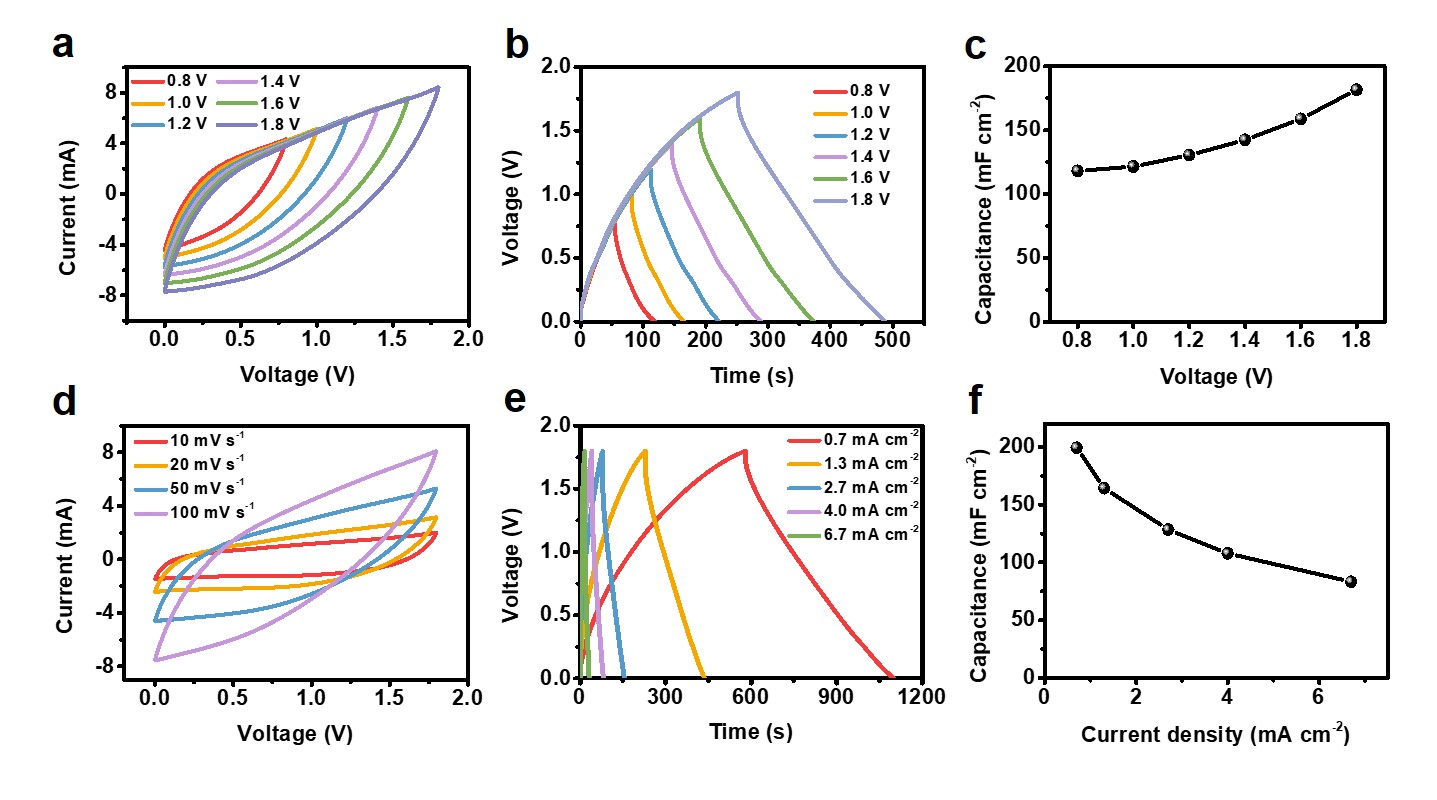


**Figure S28.** Electrochemical performance of asymmetric SC assembled by AIP+ISP. a) CV curves at different operating voltages under a scan rate of 100 mV s^-1^. b) GCD curves at different operating voltages under a current density of 1.3 mA cm^-2^. c) Areal specific capacitance obtained at a constant current density of 1.3 mA cm^-2^ as a function of the potential windows. d) CV curves at different scanning rates. e) GCD curves at different current densities. f) Areal specific capacitance obtained as a function of current density.

*
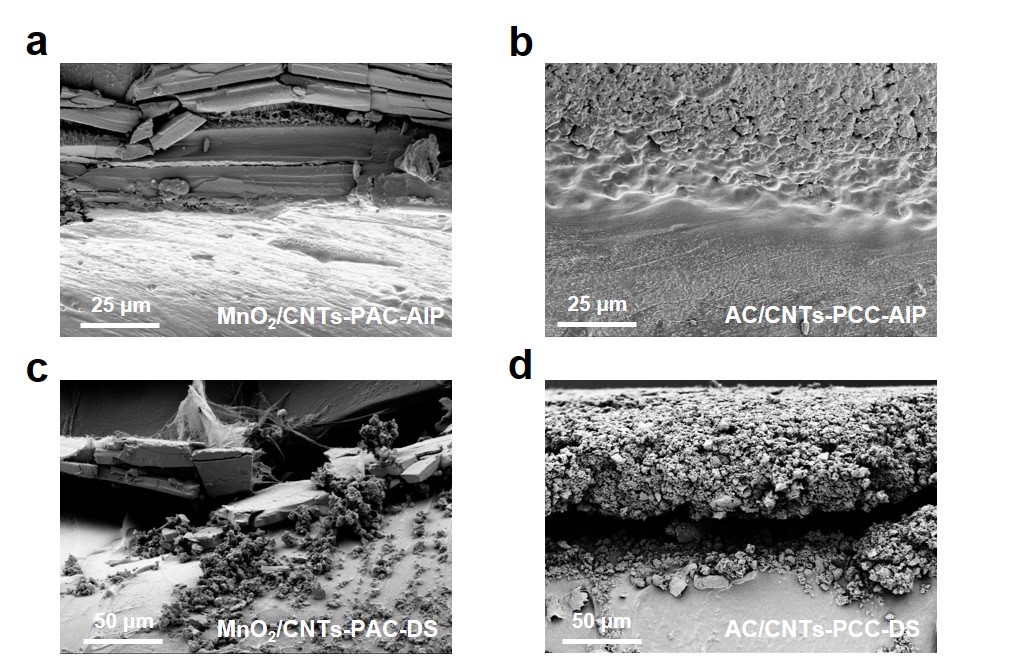
*

**Figure S29.** Cross−sectional SEM images of the interface between PAC and MnO_2_/CNTs (a), as well as between PCC and AC/CNTs (b) prepared by AIP+ISP method after 20000 charge/discharge cycles. Cross−sectional SEM images of the interface between PAC and MnO_2_/CNTs (c), as well as between PCC and AC/CNTs (d) fabricated by DS method after 20000 charge/discharge cycles.
